# The conjugated antimetabolite 5-FdU-ECyd and its cellular and molecular effects on platinum-sensitive vs. -resistant ovarian cancer cells *in vitro*

**DOI:** 10.18632/oncotarget.20260

**Published:** 2017-08-14

**Authors:** Sarah Schott, Pauline Wimberger, Barbara Klink, Konrad Grützmann, Julian Puppe, Ulrike Sophie Wauer, Daniel Martin Klotz, Evelin Schröck, Jan Dominik Kuhlmann

**Affiliations:** ^1^ Department of Gynecology and Obstetrics, University Hospital of Heidelberg, Heidelberg, Germany; ^2^ German Cancer Consortium (DKTK), Dresden and German Cancer Research Center (DKFZ), Heidelberg, Germany; ^3^ National Center for Tumor Diseases (NCT), Heidelberg, Germany; ^4^ Department of Gynecology and Obstetrics, Medical Faculty and University Hospital Carl Gustav Carus, Technische Universität Dresden, Dresden, Germany; ^5^ National Center for Tumor Diseases (NCT), Partner Site Dresden, Dresden, Germany; ^6^ Institute for Clinical Genetics, Faculty of Medicine Carl Gustav Carus, Technische Universität Dresden, Dresden, Germany; ^7^ Department of Gynecology and Obstetrics, University Hospital of Cologne, Cologne, Germany

**Keywords:** ovarian cancer, 5-FdU-ECyd, duplex-prodrugs, TAS106, platinum-resistance

## Abstract

**Background:**

Resistance to platinum-based chemotherapy is a clinical challenge in the treatment of ovarian cancer (OC) and limits survival. Therefore, innovative drugs against platinum-resistance are urgently needed. Our therapeutic concept is based on the conjugation of two chemotherapeutic compounds to a monotherapeutic pro-drug, which is taken up by cancer cells and cleaved into active cytostatic metabolites. We explore the activity of the duplex-prodrug 5-FdU-ECyd, covalently linking 2'-deoxy-5-fluorouridine (5-FdU) and 3'-C-ethynylcytidine (ECyd), on platinum-resistant OC cells.

**Methods:**

*In vitro* assays and RNA-Sequencing were applied for characterization of 5-FdU-ECyd treated platinum-sensitive A2780 and isogenic platinum-resistant A2780cis and independent platinum-resistant Skov-3-IP OC cells.

**Results:**

Nano molar 5-FdU-ECyd concentrations induced a rapid dose-dependent decline of cell viability in platinum-sensitive and -resistant OC cells. The effect of 5-FdU-ECyd was accompanied by the formation of DNA double strand breaks and apoptosis induction, indicated by a strong increase of pro-apoptotic molecular markers. Moreover, 5-FdU-ECyd efficiently decreased migration of platinum-resistant OC cells and inhibited clonogenic or spheroidal growth. Transcriptome analysis showed early up-regulation of *CDKN1A* and *c-Fos* in both, platinum-resistant and -sensitive cells after 5-FdU-ECyd treatment and de-regulation of distinct cellular pathways involved in cell cycle regulation, apoptosis, DNA-damage response and RNA-metabolism. Combined treatment of 5-FdU-ECyd and cisplatin did not show a synergistic cellular response, suggesting the potential use of 5-FdU-ECyd as a monotherapeutic agent.

**Conclusion:**

Our data provide novel mechanistic insight into the anti-tumor effect of 5-FdU-ECyd and we hypothesize that this duplex-prodrug could be a promising therapeutic option for OC patients with resistance to platinum-based chemotherapy.

## INTRODUCTION

Ovarian cancer (OC) is the leading cause of death among female gynecologic malignancies. Its mortality rate has changed only marginally over the past 30 years with a 5-year survival rate of <45 % [1]. Unfortunately, the majority of all OC patients (approx. 70 %) already present with advanced disease. In such cases, the 5-year survival rate reaches less than 20 %, due to limited therapeutic options [2, 3]. Standard treatment of OC constitutes primary tumor surgery, aiming at complete macroscopic tumor resection, followed by subsequent platinum- and paclitaxel-based chemotherapy [4]. Despite improved primary radical surgery and the implementation of innovative targeted therapy into standard treatment, such as antiangiogenetic therapy with bevacizumab or PARP-inhibitors, more than 50 % of patients suffer from recurrent disease, resulting in dismal overall prognosis [5–7]. Primary resistance to platinum-based chemotherapy, with a clinically relevant relapse within 6 months after a platin-based treatment, is observed in about 15-20 % of patients. In addition to residual postoperative tumor load as one of the most important prognostic factors, platinum-resistance, either among first line treatment or among recurrent disease, constitutes one of the most prominent clinical challenges for OC [8, 9]. Currently, neither predictive biomarkers nor adequate therapeutic concepts for patients, suffering from platinum-resistant OC, exist. Therefore, the development of innovative therapies for OC patients is urgently needed.

Our developed concept to improve polychemotherapeutic regimes aims at reversible covalent conjugation of two synergistic or additive antimetabolites, resulting in a monotherapeutic duplex-prodrug, which is metabolized into active cytotoxic compounds in tumor cells. The duplex-prodrug, investigated herein, is composed of the antimetabolites 2'-deoxy-5-fluorouridine (5-FdU) and 3'-C-ethynylcytidine (ECyd), which are covalently linked via a neutral 3'→5' diester bonding, resulting in the hetero-dinucleosidephosphate analogue 5-FdU-ECyd [10]. Intracellular metabolization of the duplex-prodrug 5-FdU-ECyd results in active cytostatic compounds that simultaneously inhibit DNA and RNA synthesis [11]. The antitumor potential of 5-FdU-ECyd is mainly based on the highly cytotoxic activity of the ECyd compound. 5-FdU is assumed to modulate the sensitivity and resistance of tumor cells [11]. Our previous studies have shown a very high nano molar efficacy of 5-FdU-ECyd in several cancer entities *in vitro* or *in vivo*, such as in hepatoblastoma, melanoma, gastric or cervical cancer [11–15]. This prodrug concept encompasses the benefits of a polychemotherapeutic regime, while avoiding typical disadvantages of single compounds, such as side effects due to higher dosing, drug resistance or premature drug metabolic breakdown. Beside some preliminary investigations [11], only very little is known about its efficacy and mechanism of action in OC cells, especially, with regard to platinum-resistance.

Therefore, the objective of the current study was to systematically characterize the effect of 5-FdU-ECyd in platinum-sensitive and platinum-resistant OC cells on a cellular and molecular level, in order to assess its potential use as a therapeutic agent for platinum-resistant OC patients.

## RESULTS

### 5-FdU-ECyd induces apoptosis and autophagy in platinum-sensitive and platinum-resistant ovarian cancer cells

**Figure 1:**
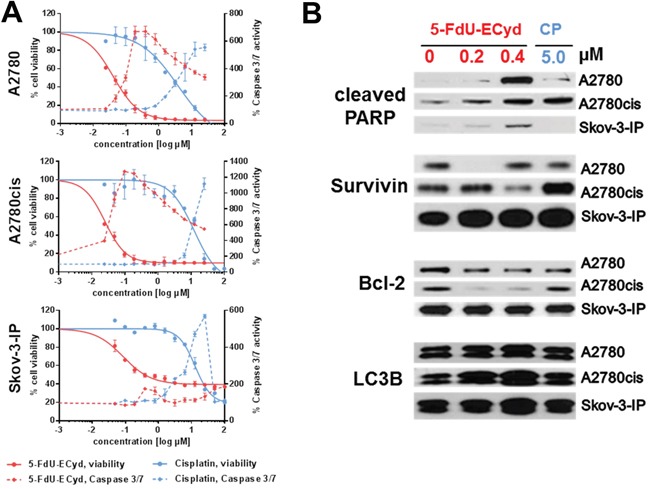
The activity of 5-FdU-ECyd in platinum-sensitive and platinum-resistant ovarian cancer cells **(A)** The figure shows viability dose-response curves (continuous lines) and caspase 3/7 activity (dashed line) in platinum-sensitive A2780 cells and platinum-resistant A2780cis/Skov-3-IP cells, following 5-FdU-ECyd (red lines) or cisplatin (blue lines) treatment. Normalized percentages of cell viability and caspase 3/7 activity were averaged from three independent experiments and are reported as mean ± SD. **(B)** Western blot analysis of A2780, A2780cis and Skov-3-IP cells, following 5-FdU-ECyd or cisplatin treatment for 48 h. The appearance of a PARP-cleavage product (89 kDa) was analyzed as pro-apoptotic marker, whereas the expression of Bcl-2 (27 kDA) and survivin (16 kDa) served as anti-apoptotic markers. The expression of LC3B was probed as marker for autophagy and appears as a double band at 14 kDa and 16 kDA.

In order to study the effect of FdU-ECyd on platinum-resistant OC *in vitro*, we took advantage of isogenic platinum-sensitive and platinum-resistant OC cells and highly malignant platinum-resistant Skov-3-IP cells for independent validation. In platinum-sensitive A2780 cells, cisplatin induced a dose-dependent reduction in cell viability with a half maximal inhibitory concentration (IC_50_) of 3.81 μM. A2780cis and Skov-3-IP cells responded less to cisplatin with an IC_50_ of 12.40 μM and 12.75 μM, respectively; confirming their resistant phenotype (Figure 1A).

After short term incubation (48 h), FdU-ECyd treatment of OC cells conferred a rapid dose-dependent decline of cell viability in platinum-sensitive A2780 and platinum-resistant A2780cis cells, suggesting that the effect of 5-FdU-ECyd is independent from platinum-resistance status (Figure 1A). Response to 5-FdU-ECyd was ~95-fold higher in A2780 cells (IC_50_ 5-FdU-ECyd 0.04 μM vs. IC_50_ cisplatin 3.81 μM) and ~620-fold higher in A2780cis cells (IC_50_ 5-FdU-ECyd 0.02 μM vs. IC_50_ cisplatin 12.4 μM), compared to equimolar cisplatin concentrations.

The effect of 5-FdU-ECyd was accompanied by decreased confluency and morphological signs of apoptosis, such as cell fragmentation (Supplementary Figure 1). On the molecular level, the apoptotic phenotype of 5-FdU-ECyd treated cells was confirmed by a rapid induction of caspase 3/7 activity in A2780 and A2780cis cells, reaching its maximum at lower concentrations compared to treatment with equimolar cisplatin (Figure 1A). Moreover, we observed a dose-dependent increase in PARP-cleavage, induced by nano molar 5-FdU-ECyd, which was similar or even stronger, compared to treatment with ~12-fold higher cisplatin concentration (Figure 1B). Particularly in platinum-resistant A2780cis cells, 5-FdU-ECyd treatment reduced the expression of the anti-apoptotic proteins survivin and Bcl-2. The effect of 5-FdU-ECyd on platinum resistant Skov-3-IP cells was less pronounced, with only modest caspase 3/7 induction or PARP-cleavage and only a slight decrease in Bcl-2 expression. This was in line with the fact that this cell line expressed a high basal level of anti-apoptotic survivin, which was further increased after treatment with 5-FdU-ECyd or cisplatin, indicating a highly chemo-resistant character of these cells (Figure 1B, Supplementary Figure 2). However, 5-FdU-ECyd was still ~140-fold more efficient in eradicating these highly malignant resistant OC cells than equimolar cisplatin (IC_50_ 5-FdU-ECyd: 0.09 μM vs. IC_50_ cisplatin: 12.75 μM, Figure 1A). Besides the pro-apoptotic effect, nano molar 5-FdU-ECyd treatment induced a moderate and dose-dependent increase in autophagy in all cell lines, indicated by increased LC3B protein expression (Figure 1B, Supplementary Figure 2), which was not observed after treatment with ~12-fold higher cisplatin concentration. There was no evidence of primary necrosis after 5-FdU-ECyd treatment (Supplementary Figure 3).

Our data support that FdU-ECyd is more potent than cisplatin *in vitro* and efficiently induces apoptosis in platinum-sensitive and platinum-resistant OC cells.

### 5-FdU-ECyd inhibits tumor-associated cellular functions of platinum-resistant ovarian cancer cells

**Figure 2:**
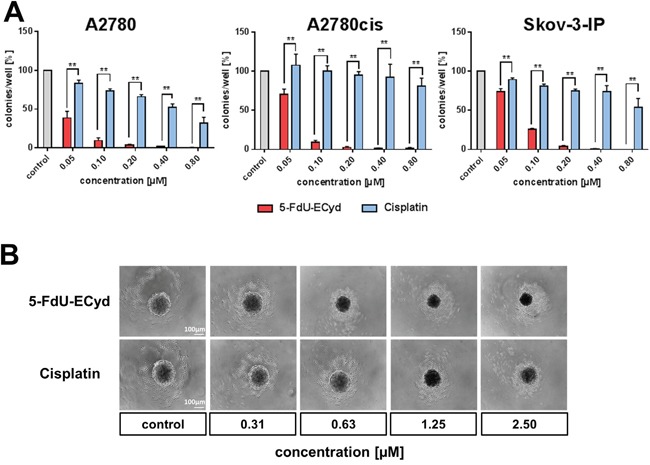
The effect of 5-FdU-ECyd on clonogenic and spheroidal growth of ovarian cancer cells **(A)** The bar chart shows the clonogenic growth of platinum-sensitive A2780 and platinum-resistant A2780cis or Skov-3-IP ovarian cancer cells, following treatment with a broad range of 5-FdU-ECyd concentrations (red bars) or equimolar cisplatin (blue bars). Normalized percentages were averaged from three independent experiments and are reported as mean ± SD. Statistical significance test, according to the unpaired t-test, resulted in a p-value ≤ 0.01 (**) among all comparisons. **(B**) The figure shows representative images (from three independent experiments) of PA-I ovarian cancer spheroid destruction, following treatment with 5-FdU-ECyd for 72 h or equimolar cisplatin, compared to untreated control.

We performed colony formation assays, in order to study the long-term effect of 5-FdU-ECyd on clonogenic growth of OC cells. 5-FdU-ECyd potently inhibited clonogenic growth in platinum-sensitive A2780 cells in the nano molar range with an almost complete eradication of colony formation at 200 nM 5-FdU-ECyd. Moreover, in isogenic A2780cis platinum-resistant cells, 5-FdU-ECyd showed similar inhibition of clonogenic growth, whereas equimolar cisplatin had virtually no effect. All results were independently confirmed in platinum-resistant Skov-3-IP cells (Figure 2A).

Subsequently, we tested, whether 5-FdU-ECyd interferes with 3-dimensional spheroidal growth *in vitro*. Since neither A2780/A2780cis cells nor Skov-3-IP cells show spheroidal growth *in vitro* at all, we applied a spheroid model system using PA-1 OC cancer cells, which form stable spheroidal aggregates with a regular membrane-like structure under serum free and low attachment conditions. This model system allows studying the effect of a given drug on spheroidal growth. Nano molar concentrations of 5-FdU-ECyd were sufficient to substantially disturb the integrity of established spheroids after 72 h incubation, indicated by disintegration of the membrane-like shape. At a concentration ≥ 1.25 μM FdU-ECyd, a complete collapse of spheroidal structures was observed. In comparison, cisplatin was also able to destroy established spheroids; however, this occurred only after treatment with ~2-fold higher micro molar concentrations (Figure 2B).

**Figure 3:**
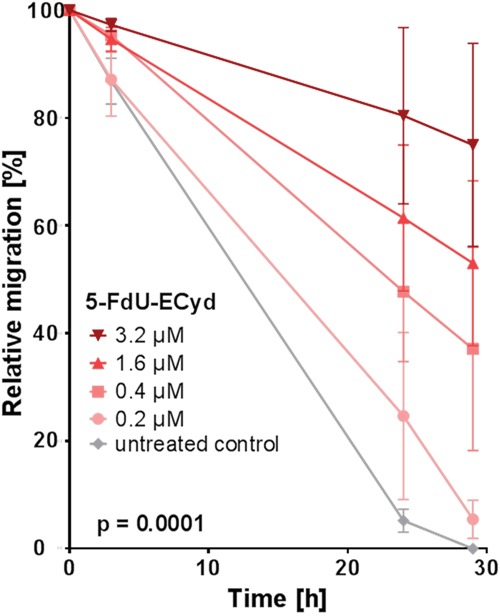
The effect of 5-FdU-ECyd on the migration of platinum-resistant Skov-3-IP cells The figure shows the relative migration of platinum-resistant Skov-3-IP ovarian cancer cells after treatment with a broad range of 5-FdU-ECyd concentrations (colored lines), compared to untreated control cells (grey line). Relative migration was calculated by measuring the percentage of the cell free area (closure of the gap) at the indicated time points and for each treatment condition. Values were calculated from three independent experiments and are reported as mean ± SEM. Statistical significance is indicated, according to the two-way ANOVA test.

Finally, we tested, whether 5-FdU-ECyd influences migration and invasion of platinum-resistant OC cells. For this purpose, platinum-resistant Skov-3-IP cells were applied, due to their strong endogenous migration characteristics *in vitro*, which can be reduced by e.g. combined PI3K- and PARP-inhibition as an appropriate positive control for this assay (Supplementary Figure 4). 5-FdU-ECyd significantly inhibited migration of Skov-3-IP cells in a time- and dose-dependent manner (p=0.0001), reaching a ~40 % migration reduction (of morphologically viable cells) after 29 h incubation at 0.4 μM 5-FdU-ECyd, compared to the untreated control (Figure 3). After treatment with ≥ 1.6 μM 5-FdU-ECyd, Skov-3-IP cells lost most of their migration capacity; however this was accompanied by morphological signs of progressive apoptosis.

To conclude, we demonstrate that 5-FdU-ECyd is a potent inhibitor of clonogenic growth and migration, independently of platinum-resistance. Moreover, according to our *in vitro* spheroid model system, nano molar 5-FdU-ECyd also inhibits 3-dimensional spheroidal growth.

### 5-FdU-ECyd induces double strand brakes

**Figure 4:**
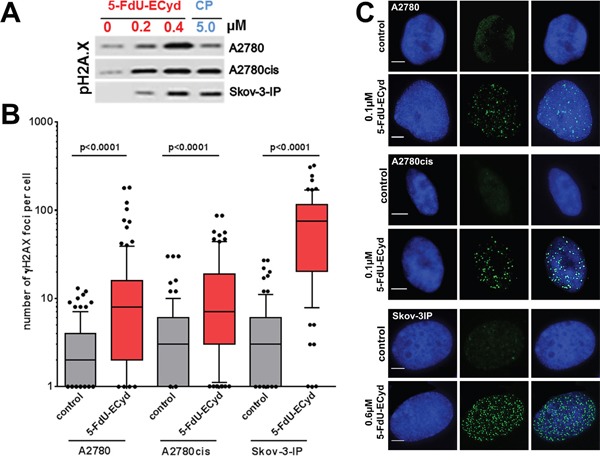
DNA double strand break analysis in 5-FdU-ECyd treated cells **(A)** Western blot analysis, analyzing phosphorylated histone H2AX in A2780, A2780cis and Skov-3-IP cells, following treatment with 5-FdU-ECyd or cisplatin. **(B)** Quantification of immunofluorescent γH2AX foci analysis in platinum sensitive A2780 and platinum-resistant A2780cis cells, following treatment with 5-FdU-ECyd (A2780 and A2780cis: 0.1 μM; Skov-3-IP 0.4 μM) for 48 h. The detection of γH2AX foci is a surrogate marker for DNA double strand brakes. γH2AX foci were counted and averaged across a hundred randomly selected cells per condition and are plotted along a logarithmic scale (log10). The whiskers are drawn down to the 10th percentile and up to the 90th. Points below and above the whiskers are drawn as individual dots. The line in the middle of the box is plotted at the median. **(C)** Representative immunofluorescent images of double strand break analysis with nucleic staining (Hoechst 33342, blue, first row), γH2AX foci staining (Alexa Flour 488, green, second row) and merged images (third row).

The most commonly described effect of platinum-based chemotherapeutics is the induction of DNA-damage in form of e.g. DNA-crosslinks or double strand breaks (DSBs), followed by the activation of DNA-damage response pathways and apoptosis induction [16–18]. Considering an interaction of 5-FdU-ECyd with DNA metabolism, we investigated, whether the conjugate duplex-prodrug is able to induce DSBs in OC cells. Western blot analysis indicated that nano molar 5-FdU-ECyd causes a dose-dependent phosphorylation of histone H2AX in the investigated OC cell lines, which is a well described surrogate marker for DSB formation [19] (Figure 4A, Supplementary Figure 5). Of note, DSB induction by nano molar 5-FdU-ECyd was similarly efficient or stronger, compared to the effect observed in OC cells, which were treated with ~10-fold higher concentrated cisplatin. Immunofluorescence analysis confirmed these findings and demonstrated a highly significant increase in the average number of γH2AX foci per cell in 5-FdU-ECyd treated A2780, A2780cis and Skov-3-IP cells, compared to untreated controls (Figure 4B, 4C).

Considering that the number of γH2AX foci per 5-FdU-ECyd treated cell had a wide scatter, especially in Skov-3-IP cells, we supposed that 5-FdU-ECyd mediated DSB induction is cell cycle dependent. In line with this assumption, co-staining for S-phase cells with EdU revealed, that γH2AX foci were more frequently present in EdU-positive S-phase cells than in EdU-negative non-replicating cells (A2780: 75 % EdU-positive vs. 25 % EdU-negative, p = 0.028; A2780cis: 89 % EdU-positive vs. 11 % EdU-negative, p = 0.006; Skov-3-IP: 68 % EdU-positive vs. 32 % EdU-negative, p = 0.006, Supplementary Figure 6).

In summary, we demonstrate that 5-FdU-ECyd induces DSBs in OC cells, independently of platinum-resistance status and preferably during DNA-replication in S-phase cells. Of note, FdU-ECyd induces DSBs more efficiently than cisplatin.

### Combined 5-FdU-ECyd and cisplatin treatment does not induce a synergistic cellular response in platinum-resistant ovarian cancer cells

**Figure 5:**
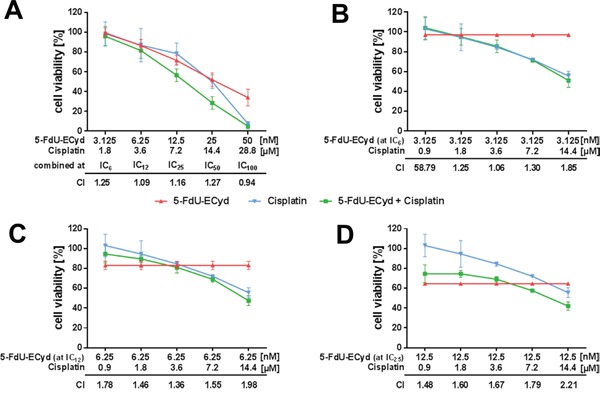
Drug interaction analysis of 5-FdU-ECyd and cisplatin **(A)** The figure shows cell viability dose-response curves for platinum-resistant A2780cis cells treated with 5-FdU-ECyd alone (red curve), cisplatin alone (blue curve) and both drugs combined (green curve). In this experiment, drugs were combined at five equipotent molar concentrations (IC_6_: 5-FdU-ECyd 3.125 nM + cisplatin 1.8 μM; IC_12_: 5-FdU-ECyd 6.25 nM + cisplatin 3.6 μM; IC_25_: 5-FdU-ECyd 12.5 nM + cisplatin 7.2 μM; IC_50_: 5-FdU-ECyd 25 nM + cisplatin 14.4 μM; IC_100_: 5-FdU-ECyd 50 nM + cisplatin 28.8 μM). For each combination, a combination index (CI) value was calculated and is indicated in the diagram, in order to describe drug interaction over the entire molar range of drug combinations. A CI-value < 0.9 indicates a synergistic interaction, a CI-value between 0.9 and 1.1 indicates an additive interaction and a CI-value > 1.1 indicates an antagonistic interaction [43]. **(B-D)** The figures show standard cell viability dose-response curves for cisplatin with a fixed concentration of low dose 5-FdU-ECyd as additional drug (green curves, 5-FdU-ECyd at IC_25_: 3.125 μM or IC_12_: 6.25 μM or IC_6_: 12.5 μM), compared to cisplatin alone (blue curves) and 5-FdU-ECyd alone (red curves). In all experiments, normalized percentages of cell viability were averaged from three independent experiments and are reported as mean ± SD.

A previous study showed that ECyd as monotherapeutic increases the effect of cisplatin in platinum-resistant cancer cells, in terms of a potential cisplatin-sensitizer [20]. Therefore, we were interested in studying possible *in vitro* drug interactions between the duplex-prodrug 5-FdU-ECyd and cisplatin in platinum-resistant A2780cis OC cells. In a first approach, we studied the interaction of 5-FdU-ECyd and cisplatin, when combined at a broad range of six equipotent molar concentrations (calculative at: IC_6_, IC_12_, IC_25_ IC_50_ and IC_100_). An additive effect was observed among 2 of 5 concentration levels (at IC_12_ and IC_100_; 0.9 < CI < 1.1). Among the three remaining combinations (IC_6_, IC_25_, IC_50_) the observed drug interaction was lower than an expected additive interaction and was consequently classified as a “slight to moderate antagonistic interaction” (CI-value > 1.1, Figure 5A).

Considering the strong activity of 5-FdU-ECyd in OC cells at very low nano molar concentrations and the at least partially additive interaction between 5-FdU-ECyd and cisplatin, we further inquired, whether the effective dose for a cisplatin response in platinum-resistant A2780cis cells could be reduced by adding low dose 5-FdU-ECyd. We compared standard cisplatin dose-response curves, while keeping 5-FdU-ECyd at three different (fixed) low dose concentrations (calculative at: IC_6_ or IC_12_ or IC_25_ 5-FdU-ECyd, Figure 5B–5D). The interaction between 5-FdU-ECyd and cisplatin was lower than an expected additive interaction in nearly all cases (CI > 1.1) and was therefore classified as “antagonistic”.

Our data demonstrate the lack of a synergistic interaction between 5-FdU-ECyd and cisplatin, suggesting its potential utility as a monotherapeutic drug in platinum-resistant OC, rather than a cisplatin-sensitizer.

### Transcriptome analysis of 5-FdU-ECyd treated ovarian cancer cells

**Figure 6:**
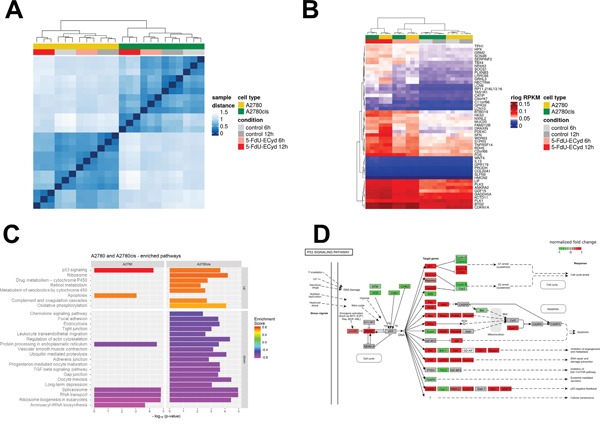
Transcriptome analysis of 5-FdU-ECyd treated platinum-sensitive and platinum resistant ovarian cancer cells **(A)** Heat map from hierarchical clustering of Euclidian sample distances based on regularized logarithmic (rlog) transformed expression values of all genes of all samples. The top color bar represents the cell line and the second color bar the treatment type. Each row and column denotes a sample. The dendrogram on the x-axis shows the grouping of samples according to biological replicates (triplicates), treatment and cell type. **(B)** Heat map showing hierarchical clustering based on 50 top ranking differentially expressed genes between 5-FdU-ECyd treated and untreated cells in A2780 and A2780cis (rlog transformed data). The top color bar represents the cell line and the second color bar the treatment type. Each row denotes a single gene and their expression pattern across the samples. The dendrogram on the x-axis shows the grouping of same samples. The two main groups are defined by treated and untreated cells (red and grey columns), and within those groups further separating according to cell type (A2780 in yellow and A2780cis in green) and biological replicates (triplicates). **(C)** Bar chart shows commonly or uniquely enriched pathways after short term 5-FdU-ECyd exposure (1 μM for 12 h) of platinum-sensitive (A2780) and platinum-resistant (A2780cis) ovarian cancer cells. Bar lengths correspond to -log_10_(p-value) of the enrichment hypothesis test, while bar colors represent enrichment scores (ES). Pathways are vertically sorted by ES and thereby divided into up-regulated (ES>0) and down-regulated (ES<0) ones. **(D)** Figure showing deregulation of the p53-pathway following 5-FdU-ECyd treatment of A2780 cells. Normalized log_2_ FCs after 12 h treatment are color coded. KEGG pathways were plotted using the R package pathview [44].

To understand its mechanism of action, we studied the effect of 5-FdU-ECyd treatment on the global gene expression profile of OC cells in relation to platinum-resistance status. Therefore, triplicate samples of platinum-sensitive A2780 and isogenic platinum-resistant A2780cis cells were subjected to short term 5-FdU-ECyd exposure (1 μM for 6 h or 12 h), at which no morphological signs of cell death were detected yet, followed by RNA isolation and subsequent whole transcriptome sequencing. Sample distance analysis, hierarchical clustering and principal components analysis showed a clear separation between A2780 and A2780cis in two groups and a strong effect of 5-FdU-ECyd exposure after 12 h within each cell line, while there was only a mild effect after 6 h treatment (Figure 6A; Supplementary Figures 7 and 8). Gene expression analysis revealed similar numbers of significantly differentially expressed genes (DEG, Benjamini-Hochberg adjusted-value ≤ 0.05) when comparing 5-FdU-ECyd treated vs. untreated cells (A2780: 9937 genes; A2780cis 9638 genes). The majority of DEG overlapped between both cell lines (6306 genes; Supplementary Figure 9A). We identified 515 and 643 strongly up- or downregulated genes in A2780 and A2780cis, respectively (adjusted p-value ≤ 0.05 and absolute log_2_ FC ≥ 2); most of those genes were up-regulated (435 and 505 up-regulated genes, respectively) and 177 DEG overlapped between both cell lines (Supplementary Figure 9B). After 6 h treatment, only 13 and 35 genes, respectively, were highly and significantly deregulated in A2780 and A2780cis (adjusted p-value ≤ 0.05 and absolute log_2_ FC ≥ 2). Hierarchical clustering using the 50 top ranking common DEG clearly separated untreated from treated cells (Figure 6B).

Pathway analysis using three different algorithms revealed similar results (Figure 6C). In both, platinum-sensitive and -resistant cell lines, the p53 pathway was activated after 5-FdU-ECyd exposure (Figure 6D; Supplementary Figures 10 and 11). In this context, the expression of several p53 target genes was significantly up-regulated, particularly those involved in cell cycle regulation (*CDKN1A*, *14-3-3- 029A*, *cyclin E*, *CDK2*, *GADD45*), DNA-repair (*P48*, *p53R2*, *GADD45*, *Sestrin*) and apoptosis (*Bax*, *Noxa*, *PUMA*, *Apaf-1*, Supplementary Figure 8). Interestingly, *CDKN1A* and *GADD45* were among the top ranking up-regulated genes in both cell lines (*CDKN1A:* log_2_ FC = 2.7 and 4.0 in A2780 and A2780cis; *GADD45*: log_2_ FC = 3.1 and 3.2 in A2780 and A2780cis), which were significantly up-regulated already after 6 h treatment (*CDKN1A:* log_2_ FC = 2.4 and 2.8 in A2780 and A2780cis; *GADD45*: log_2_ FC = 1.3 and 2.0 in A2780 and A2780cis). Consistent with the fact that 5-FdU-ECyd treatment was performed no longer than 12 h, final effector enzymes of apoptosis, such as caspase 3, were not yet differentially expressed in 5-FdU-ECyd treated OC cells. 5-FdU-ECyd treatment additionally affected RNA-related housekeeping pathways and significantly decreased the expression of non-coding small nuclear RNAs and coding RNAs of the spliceosome in both, platinum-sensitive and platinum-resistant cells (Figure 6C). Moreover, it affected RNA-transport and inhibited ribosome biogenesis pathways (Supplementary Figures 12 and 13). Transcripts associated with protein processing in the endoplasmatic reticulum were also down-regulated in 5-FdU-ECyd treated cells. In platinum-sensitive A2780 cells, a slightly broader spectrum of apoptosis related transcripts were up-regulated, including genes of death receptor, NF-kB or TNF-associated pathways (e.g. *TRAIL*, *Fas*, *TNFa*, *IKK*, *IkBa*). In platinum-resistant A2780cis cells, on the other hand, additional pathways involved in drug metabolism or oxidative phosphorylation were significantly up-regulated, especially cytochrome P450-associated pathways. Interestingly, in both cell lines, a strong up-regulation of *c-Fos* was observed after 12 h treatment (log_2_ FC = 2.6 and 3.9 in A2780 and A2780cis), and *c-Fos* was also amongst the genes significantly up-regulated already after 6 h (log_2_ FC = 1.6 and 2.7 in A2780 and A2780cis).

In summary, 5-FdU-ECyd leads, independently of platinum-resistance status, to an activation of genes and pathways involved in cell cycle, apoptosis, and DNA-damage response, while it downregulates RNA-related pathways. Interestingly, we found early and strong up-regulation of *c-Fos* oncogene after exposure to 5-FdU-ECyd. Moreover, differential activation of pathways related to energy homeostasis and drug metabolism, was observed only in platinum-resistant OC cells.

## DISCUSSION

In the present study, we characterized the effect of the duplex-prodrug 5-FdU-ECyd in OC cells *in vitro* on a phenotypic and molecular level with particular focus on platinum-resistance.

Previous *in vitro* studies suggested a highly sensitive effect of 5-FdU-ECyd towards OC cells as well as other cancer cell types [10, 11, 15]. Herein, we reported that 5-FdU-ECyd confers a rapid dose-dependent apoptotic response of platinum-sensitive OC cells, clearly exceeding the effect of cisplatin. Moreover, in (isogenic or independent) platinum-resistant OC cells, 5-FdU-ECyd induced a similar apoptotic response, indicating that its mechanism of action is independent of platinum-resistance status. These findings are in accordance to our previous *in vitro* study on cervical cancer, reporting that nano molar 5-FdU-ECyd induces apoptotic cell death and early S-phase arrest, also including platinum-resistant SiHa cells [12]. Besides apoptosis induction, we noticed that 5-FdU-ECyd induces autophagy. Autophagy is an intracellular nutrient scavenging pathway, whereby cells “digest” parts of themselves and recycle the breakdown products, in order to sustain survival during starvation periods [21]. The observed increase in autophagy is likely part of an integrated stress response, following the 5-FdU-ECyd treatment, which may precede apoptosis induction.

We demonstrated that 5-FdU-ECyd decreases clonogenic and spheroidal growth of OC cells. Considering that spheroid formation in ascites is highly associated with chemo-resistant characteristics [22–24], our data emphasize the efficacy of 5-FdU-ECyd against platinum-resistant OC, which often goes along with tumor spread throughout the peritoneal cavity. Moreover, a rather specific inhibition on migration by 5-FdU-ECyd was shown at dosing of 200-400 nM without any signs of apoptosis, which typically occurred at higher 5-FdU-ECyd concentrations. Therefore, we suggest 5-FdU-ECyd as an effective anti-tumor agent for the elimination of OC cells *in vitro*, since it blocks not only the proliferation by inducing cell cycle arrest, but also suppresses superordinate cellular functions, such as migration at low concentrations.

There was no synergistic interaction observed between 5-FdU-ECyd and cisplatin in our study, whereas a previous study reported on a synergistic cellular response, following a combined treatment of head and neck cancer cells *in vitro* with cisplatin and ECyd as monotherapeutic [20]. This outlines the different properties of the duplex-prodrug, compared to ECyd as monotherapeutic [10]. We conclude that 5-FdU-ECyd is more potent than cisplatin and can potentially act as single regime with lower side effects, especially in case of platinum-resistance, rather than a combinational partner within a polychemotherapeutic schedule. This is an important aspect as the monotherapeutic ECyd caused critical side effects in clinical studies [25, 26].

In order to obtain a deeper insight into the activity of 5-FdU-ECyd in platinum-resistant OC cells, we performed exploratory transcriptome analysis. The fact that we detected over-expression of early response genes (e.g. *c-Fos*) and did not yet notice over-expression of apoptotic effector enzymes (e.g. *caspase 3*), indicated that our transcriptome data reflect a rather early response signature to 5-FdU-ECyd. The p53-pathway, which is a major driver of cell cycle arrest and apoptosis [27], was consistently activated in A2780 and A2780cis cells, which is in accordance to our *in vitro* experiments on 5-FdU-ECyd mediated apoptosis and is also in line with previous studies, reporting that 5-FdU-ECyd induces apoptosis and G1-arrest in cervical cancer cells *in vitro* [12]. *CDKN1A* and *c-Fos* were among the most consistently up-regulated genes, showing a significant up-regulation already after 6 h of 5-FdU-ECyd treatment. *CDKN1A* is an important p53 transcriptional target, which induces cell cycle arrest. The *c-Fos* gene encodes for the immediate early gene product Fos, which is a transcription factor of the activating protein-1 (AP1) family. Since *c-Fos* was originally described as an oncogene [28], its up-regulation during 5-FdU-ECyd mediated cytostasis/apoptosis might seem counterintuitive at first sight. However, some exploratory studies have already described an association between *c-Fos* activation and 5-FdU induced cell death [29]. Moreover, there is also evidence that *c-Fos* can exert pro-apoptotic functions in OC cells [30] and can induce cell cycle arrest by *CDKN1A* up-regulation [31]. This suggests that the conjugate duplex-drug 5-FdU-ECyd may additionally employ *c-Fos* signaling for its cytostatic and apoptotic effect on platinum-sensitive and platinum-resistant OC cells.

We observed a global decrease in RNA-housekeeping pathways in platinum-sensitive and -resistant OC cells, including RNA-transport, spliceosome and ribosome biogenesis pathways. This finding is consistent with the fact that ECyd, a potent RNA-polymerase inhibitor [20, 26], inhibits RNA-synthesis in OC cells after being cleaved from the duplex-prodrug. Furthermore, we demonstrate here that 5-FdU-ECyd induces DNA-damage in form of DSBs, which were preferentially observed in S-phase during DNA-replication. This is an interesting finding and further complements possible mechanisms, how 5-FdU-ECyd exerts its cytostatic and pro-apoptotic activity. It has previously been reported that 5'-fluorouracil (5-FU), which can be intracellularly anabolized to 5-FdU, induces DSBs *in vitro* [32]. After being released from the duplex-prodrug, we believe that 5-FdU inhibits DNA-synthesis [33] and may also induce DNA-DSBs in the OC investigated herein. Therefore, we propose that, among the already described effects of 5-FdU-ECyd in tumor cells [11], the induction of DNA-DSBs is a further mechanism of action, which may converge on the induction of p53-mediated apoptosis. Moreover, our pathway analysis supports the clinical utility of a polychemotherapeutic cancer treatment by a duplex-prodrug, which might particularly be essential for targeting highly heterogeneous tumors, such as OC.

The best-characterized effect of cisplatin is the induction of mitochondrial apoptosis [34]. In platinum-resistant cells, several molecular mechanisms, such as increased DNA-repair capacity or the alteration of DNA-damage associated pathways, result in apoptosis inhibition and cell survival, following cisplatin treatment [17]. In platinum-resistant A2780cis cells, we observed a 5-FdU-ECyd mediated activation of cytochrome p450 drug metabolization pathways, which are known to confer chemo-resistance [35]. Interestingly, although these pathways were activated, the antitumor effect of 5-FdU-ECyd was not impaired at all in these cells. Therefore, although 5-FdU-ECyd utilizes similar signaling pathways like cisplatin for its apoptotic effect, it seems to bypass common mechanisms of platinum-resistance. Nevertheless we suppose that the generally impaired apoptotic response of Skov-3-IP, with low caspase 3/7 induction as a surrogate marker, could be explained by a compensative activity of anti-apoptotic pathways in these cells. This assumption is supported by our western blot findings, since we observed a higher basal level of anti-apoptotic survivin and a slight survivin up-regulation in Skov-3-IP after 5-FdU-ECyd treatment. Moreover, there was a lack of anti-apoptotic Bcl-2 downregulation in Skov-3-IP. Additionally, specific resistance mechanism towards antimetabolites could also impair response towards 5-FdU-ECyd, since it is known, for instance, that decreased phosphorylation of ECyd by uridine/cytidine kinase can promote ECyd-resistance [36].

In summary, our *in vitro* study provides further insight into the activity of 5-FdU-ECyd in cancer cells. Moreover, we provide rationale to analyze this therapeutic agent in an ovarian cancer *in vivo* model, in order to further assess its potential utility in platinum-resistant ovarian cancer patients.

## MATERIALS AND METHODS

### Cell culture

Detailed information on the cell lines used are provided in Supplementary Materials. The isogenic human OC cell lines A2780 and A2780cis were purchased from Sigma-Aldrich (Taufkirchen, Germany). Re-authentication of cell lines was successfully performed before the onset of the experiments by a commercial authentication service (DSMZ) using DNA-STR profiling, as soon as a public reference profile was available. Mycoplasma testing by PCR was performed weekly by a commercial in house service.

### Drugs

5-FdU-ECyd was kindly provided by H. Schott. Cisplatin was purchased from Accord Healthcare GmbH (Freilassing, Germany). BKM120 and Olaparib were purchased from Selleck Chemicals (Houston, USA).

### Detection of cell viability, caspase 3/7 activity and cytotoxicity

Cell viability, caspase 3/7 activity and cytotoxicity (primary necrosis) following drug treatment of OC cells *in vitro*, were assessed using the ApoTox-Glo™ Triplex Assay (Promega, Fitchburg, USA). This assay quantifies the activity of “live-cell protease” as an estimate for cell viability, caspase 3/7 activity as an estimate of apoptosis induction and “dead-cell protease” as an estimate of cytotoxicity, which corresponds to primary necrosis. Briefly, OC cells were seeded at a density of 10.000 cells/well in a 96-well plate. Cells were incubated for 24 h in standard cell culture medium, subsequently incubated for 48 h with drug-containing medium and finally assayed, according to the manufacturer's instructions. Cell viability and cytotoxicity were detected with a fluorescence reader (Infinite M200, Tecan, Männedorf, Switzerland) at 505 nm or 520 nm, respectively. Caspase 3/7 activity was quantified by a luminescence reader (Microplate Luminometer LB96 V, EG&G Berthold, Bad Wildbad, Germany). All experiments were performed in technical triplicates and results were averaged from three independent experiments. Log(Dose) vs. response curves (variable slope) were drawn with Prism 6.04 (GraphPad Software, CA, USA).

### Western blot analysis

A2780, A2780cis and Skov-3-IP cells were grown to 80-90 % subconfluency, trypsinized, lysed in RIPA Lysis Buffer (Santa Cruz, Dallas, USA) and shaked for 30 min with 350 rpm at 4 °C in order to prepare whole cell lysates. Subsequently, 20 μg whole cell lysate per sample were subjected to a 4-12 % Bis-Tris protein gel (Thermo Fisher Scientific) and transferred onto nitrocellulose (NC) membranes (Amersham^TM^ Protran^TM^ Premium 0.45 μm NC, GE Healthcare Life science, Chalfont St Giles, UK). After protein transfer, membranes were blocked overnight in 1xPBS/ 0.1 % Tween20 (PBS-T), supplemented with 5 % (w/v) skimmed milk. Subsequently, (dissected) NC-membranes were incubated with anti-ß-actin (AC-74) antibody (Sigma-Aldrich, Taufkirchen, Germany; 1:10^5^ in PBS-T), anti-survivin (71G4B7) antibody (Cell Signaling Technology, Boston, USA; 1:10^4^ in PBS-T), anti-Bcl-2 (C-2) antibody (Santa Cruz; 1:100 in PBS-T), anti-cleaved PARP (Asp214) (D64E10) antibody (Cell Signaling Technology; 1:500 in PBS-T) anti-LC3B (D11) antibody (Cell Signaling Technology; 1:500 in PBS-T) or primary rabbit-anti-phospho-pH2A.X antibody (Cell Signaling Technology) for 1 h at room temperature. Membranes were incubated for detection with secondary antibodies, raised against rabbit and linked with HRP (Cell Signaling Technology) or raised against mouse and linked with HRP (Dianova, Hamburg, Germany) for 1 h at room temperature.

### Colony formation assay

Colony formation assays were applied, in order to characterize 2-dimensional clonogenic growth of OC cells after treatment with cytotoxic agents. Therefore, cells were plated in a 6-well format at very low density (500 cells per 3 ml cell culture medium per well), allowing each single cell to grow into a colony. After 48 h, cells were incubated with 5-FdU-ECyd or cisplatin containing medium at the indicated concentrations for 6 d, fixed (10 % formaldehyde, 2 ml per well, 30 min) and stained with 0.05 % crystal violet 2 ml per well, 30 min). Resulting colonies were counted under a light microscope. A focal accumulation of at least 50 stained cells was considered a “cell colony”. All experiments were performed in technical duplicates and colony counts were averaged from three independent experiments. Statistical analysis was performed with Prism 6.04 (GraphPad Software, CA, USA), using the paired t-test.

### Spheroid destruction assay

Spheroid destruction assays were applied, in order to characterize 3-dimensional spheroidal growth of OC cells after drug treatment. For this assay, PA-I OC cells were applied, which form stable spheroidal aggregates (with a regular membrane-like structure) under serum free and low attachment conditions. Briefly, 96-well plates were prepared by adding 50 μl 2 % (w/v) agarose solution per well, which solidifies during incubation at 37 °C for 30 min. Subsequently, PA-I OC cell were seeded with a density of 500 cells/well in 100 μl culture medium. After 3-4 days, when stable spheroids had formed and reached their final diameter, drug containing medium was added for 72 h. The effect of drug treatment on spheroidal structures was monitored every 24 h by light microscopy. All drug concentrations were tested on OC spheroids in technical triplicates and were repeated among three independent experiments.

### Immunofluorescent staining

For γH2AX/EdU-immunofluorescence analysis, 40.000 cells/well were seeded on coverslips in a 24-well plate and cultured for 24 h in standard cell culture medium, followed by incubation with 5-FdU-ECyd containing medium (0.1 μM 5-FdU-ECyd for A2780 and A2780cis; 0.4 μM for Skov-3-IP) for 48 h. Subsequently, cells were treated for 2 h with 10 μM EdU (Thermo Fisher Scientific) and fixed in 2 % formaldehyde. For γH2AX staining, samples were incubated with a primary rabbit-anti-phospho-pH2A.X antibody (Cell Signaling Technology) overnight at 4 °C and with an Alexa Fluor 488–conjugated secondary antibody (Dianova) for 90 min at room temperature. For EdU staining, the Click-iT® EdU Alexa Fluor® 555 Imaging Kit (Thermo Fisher Scientific) was used following the manufacturer's instructions. The quantitative analysis of γH2AX foci is described in Supplementary Materials.

### Migration assay

Migration assays were performed by the commercially available μ-Slide 8 Well Grid-500 system (Ibidi, Martinsried, Germany), according to the manufacturer's instruction. This system is a standardized variant of the manual scratch assay, which allows setting a standardized gap in a confluent monolayer of cells, which can subsequently be monitored for continuous occupation by migrating cells. Briefly, 14.000 cells were seeded per chamber in a volume of 70 μl standard growth medium or 5-FdU-ECyd containing medium at the indicated concentrations. After 24 h incubation, inserts were removed and cells were incubated in drug-free standard growth medium for a total time period of 29 h, allowing them to migrate into the gap. Microscopic assessment of gap closure was performed directly after medium change (baseline) and at 3 h, 24 h and 29 h. Relative migration was calculated by measuring the percentage of the cell free area in each condition, compared to untreated control cells. As a positive control for this assay, Skov-3-IP cells were treated with 1 μM BKM120 (Selleck Chemicals, Houston, USA) and 2 μM Olaparib (Selleck Chemicals) after insert removal, resulting in a significantly reduced migration up to 40 % (p=0.001, Supplementary Figure 4). Statistical analysis was performed with Prism 6.04 (GraphPad Software, CA, USA), using the two-way analysis of variance (ANOVA) test.

### RNA isolation and whole transcriptome sequencing

For Whole-Transcriptome Sequencing (RNA-Seq), 2×10^6^ cells (A2780) or 2.5×10^6^ cells (A2780cis) were seeded in T-75 flasks. After 48 h, medium was removed and replaced with 1 μM 5-FdU-ECyd containing medium or standard growth medium as control. After 6 h or 12 h drug treatment, cells were harvested for RNA extraction, according to the miRNeasy Mini Kit (Qiagen, Hilden, Germany). On column DNA digestion was included into this protocol, in order to remove residual contaminating genomic DNA. All experiments were done in triplicates and according to the manufacturer's instructions. For library preparation we used the TruSeq Stranded Total RNA Library Prep Kit (Illumina) according to the manufacturer's protocol, starting with 1 μg total RNA. All barcoded libraries were pooled and sequenced 2×75bp paired-end on an Illumina NextSeq500 platform to obtain a minimum of 10 Mio reads per sample.

### Bioinformatic analysis

Detailed information about the bioinformatic analysis is described in Supplementary Materials. Briefly, reads were trimmed using trimmomatic [37] and aligned using STAR [38]. Read counts were extracted from the alignments using featureCounts method of the Rsubread package [39]. DESeq2 was applied to identify differentially expressed genes using standard parameter settings [40]. Only genes with multiple testing adjusted p-values (padj from DESeq2) < 0.05 were considered significant.

Pathway analysis was performed using three different approaches. First, Gene Ontology (GO) and KEGG enrichment of differentially expressed gene lists were calculated using DAVID Bioinformatics Resource [41]. Secondly, the R package gage was applied on the log_2_ fold-changes (log_2_ FC) of all genes for the different conditions using KEGG pathways [42]. Thirdly, the R package fgsea was used for a full gene set enrichment analysis. Log_10_ (p-value) * signum(log_2_ FC) was used as rank function and 100,000 permutations for p-value calculation of pathway enrichments.

### Data availability

Raw data files and quantified gene expression information (as described above) have been submitted to Gene Expression Omnibus (GEO) with the accession number GSE98230.

### Drug interaction analysis

Drug interaction analysis between 5-FdU-ECyd and cisplatin was based on cell viability data, obtained by the fluorometric Cell Titer Blue^®^-Assay (Promega, Fitchburg, USA), according to the manufacturer's instructions. Statistical analysis was performed by the combination index method [43], which is based on the combination of drugs at a broad range of equipotent molar concentrations or, as our modification, with standard cisplatin dose-response curves with a fixed 5-FdU-ECyd concentrations at each reading point. Additional information to this method is provided in Supplementary Materials.

## SUPPLEMENTARY MATERIALS FIGURES AND TABLES



## Abbreviations

5-FdU: 2'-deoxy-5-fluorouridine
5-FU: 5'-fluorouracil
AP1: activating protein-1
CI: combination index
DEG: differentially expressed genes
DSBs: double strand breaks
ECyd: 3'-C-ethynylcytidine
FC: fold change
GO: Gene Ontology
IC_50_: half maximal inhibitory concentration
NC: nitrocellulose
OC: ovarian cancer
PBS-T: 1xPBS/ 0.1 % Tween20

## References

[1] Siegel R, Ma J, Zou Z, Jemal A (2014). Cancer statistics, 2014. CA Cancer J Clin.

[2] Ke C, Hou Y, Zhang H, Fan L, Ge T, Guo B, Zhang F, Yang K, Wang J, Lou G, Li K (2015). Large-scale profiling of metabolic dysregulation in ovarian cancer. Int J Cancer.

[3] Pujade-Lauraine E, Hilpert F, Weber B, Reuss A, Poveda A, Kristensen G, Sorio R, Vergote I, Witteveen P, Bamias A, Pereira D, Wimberger P, Oaknin A (2014). Bevacizumab combined with chemotherapy for platinum-resistant recurrent ovarian cancer: the AURELIA open-label randomized phase III trial. J Clin Oncol.

[4] du Bois A, Quinn M, Thigpen T, Vermorken J, Avall-Lundqvist E, Bookman M, Bowtell D, Brady M, Casado A, Cervantes A, Eisenhauer E, Friedlaender M, Fujiwara K (2005). 2004 consensus statements on the management of ovarian cancer: final document of the 3rd International Gynecologic Cancer Intergroup Ovarian Cancer Consensus Conference (GCIG OCCC 2004). Ann Oncol.

[5] Martin LP, Schilder RJ (2009). Management of recurrent ovarian carcinoma: current status and future directions. Semin Oncol.

[6] Burger RA, Brady MF, Bookman MA, Fleming GF, Monk BJ, Huang H, Mannel RS, Homesley HD, Fowler J, Greer BE, Boente M, Birrer MJ, Liang SX; Gynecologic Oncology Group (2011). Incorporation of bevacizumab in the primary treatment of ovarian cancer. N Engl J Med.

[7] Ledermann J, Harter P, Gourley C, Friedlander M, Vergote I, Rustin G, Scott CL, Meier W, Shapira-Frommer R, Safra T, Matei D, Fielding A, Spencer S (2014). Olaparib maintenance therapy in patients with platinum-sensitive relapsed serous ovarian cancer: a preplanned retrospective analysis of outcomes by BRCA status in a randomised phase 2 trial. Lancet Oncol.

[8] Bookman MA (1999). Extending the platinum-free interval in recurrent ovarian cancer: the role of topotecan in second-line chemotherapy. Oncologist.

[9] Davis A, Tinker AV, Friedlander M (2014). “Platinum resistant” ovarian cancer: what is it, who to treat and how to measure benefit?. Gynecol Oncol.

[10] Schott H, Schott S, Schwendener RA (2009). Synthesis and *in vitro* activities of new anticancer duplex drugs linking 2'-deoxy-5-fluorouridine (5-FdU) with 3'-C-ethynylcytidine (ECyd) via a phosphodiester bonding. Bioorg Med Chem.

[11] Schott S, Wallwiener M, Kootz B, Seeger H, Fehm T, Neubauer H (2011). ATP chemosensitivity testing of new antitumor duplex drugs linking 3`-C-ethynylycytidine (ECyd) and 2 -deoxy-5-fluorouridine (5-FdU) in comparison to standard cytostatica and combinations thereof. Invest New Drugs.

[12] Schott S, Bruning A (2014). Induction of apoptosis in cervical cancer cells by the duplex drug 5-FdU-ECyd, coupling 2'-deoxy-5-fluorouridine and 3'-C-ethinylcytidine. Gynecol Oncol.

[13] Schott S, Niessner H, Sinnberg T, Venturelli S, Berger A, Ikenberg K, Villanueva J, Meier F, Garbe C, Busch C (2012). Cytotoxicity of new duplex drugs linking 3'-C-ethynylcytidine and 5-fluor-2'-deoxyuridine against human melanoma cells. Int J Cancer.

[14] Eicher C, Dewerth A, Ellerkamp V, Fuchs J, Schott S, Armeanu-Ebinger S (2013). Effect of duplex drugs linking 2'-deoxy-5-fluorouridine (5-FdU) with 3'-C-ethynylcytidine (ECyd) on hepatoblastoma cell lines. Pediatr Surg Int.

[15] Weinreich J, Archid R, Bajaeifer K, Hack A, Konigsrainer A, Schott TC (2014). Growth and chemosensitivity of gastric adenocarcinoma and non-malignant cell lines in response to novel anti-cancer drug combinations. Chemotherapy.

[16] Jamieson ER, Lippard SJ (1999). Structure, recognition, and processing of Cisplatin-DNA adducts. Chem Rev.

[17] Galluzzi L, Senovilla L, Vitale I, Michels J, Martins I, Kepp O, Castedo M, Kroemer G (2012). Molecular mechanisms of cisplatin resistance. Oncogene.

[18] Rezaee M, Sanche L, Hunting DJ (2013). Cisplatin enhances the formation of DNA single- and double-strand breaks by hydrated electrons and hydroxyl radicals. Radiat Res.

[19] Lobrich M, Shibata A, Beucher A, Fisher A, Ensminger M, Goodarzi AA, Barton O, Jeggo PA (2010). gammaH2AX foci analysis for monitoring DNA double-strand break repair: strengths, limitations and optimization. Cell Cycle.

[20] Fukushima H, Abe T, Sakamoto K, Tsujimoto H, Mizuarai S, Oie S (2014). 3'-ethynylcytidine, an RNA polymerase inhibitor, combined with cisplatin exhibits a potent synergistic growth-inhibitory effect via Vaults dysfunction. BMC Cancer.

[21] Guo JY, White E (2016). Autophagy, metabolism, and cancer. Cold Spring Harb Symp Quant Biol.

[22] Achilli TM, Meyer J, Morgan JR (2012). Advances in the formation, use and understanding of multi-cellular spheroids. Expert Opin Biol Ther.

[23] Ahmed N, Stenvers KL (2013). Getting to know ovarian cancer ascites: opportunities for targeted therapy-based translational research. Front Oncol.

[24] Lengyel E (2010). Ovarian cancer development and metastasis. Am J Pathol.

[25] Tsao A, Hui EP, Juergens R, Marur S, Huat TE, Cher GB, Hong RL, Hong WK, Chan AT (2013). Phase II study of TAS-106 in patients with platinum-failure recurrent or metastatic head and neck cancer and nasopharyngeal cancer. Cancer Med.

[26] Abdelrahim M, Matsuda A, Naing A (2013). TAS-106: preclinical, clinical and beyond. Oncology.

[27] Espinosa JM (2008). Mechanisms of regulatory diversity within the p53 transcriptional network. Oncogene.

[28] Durchdewald M, Angel P, Hess J (2009). The transcription factor Fos: a Janus-type regulator in health and disease. Histol Histopathol.

[29] Kakutani T, Ebara Y, Kanja K, Takahashi K, Wataya Y (1998). Activation of c-jun and c-fos genes in dNTP imbalance cell death induced with 5-fluoro-2'-deoxyuridine in mouse mammary tumor FM3A cell line. Nucleosides Nucleotides.

[30] Oliveira-Ferrer L, Rossler K, Haustein V, Schroder C, Wicklein D, Maltseva D, Khaustova N, Samatov T, Tonevitsky A, Mahner S, Janicke F, Schumacher U, Milde-Langosch K (2014). c-FOS suppresses ovarian cancer progression by changing adhesion. Br J Cancer.

[31] Chan QK, Lam HM, Ng CF, Lee AY, Chan ES, Ng HK, Ho SM, Lau KM (2010). Activation of GPR30 inhibits the growth of prostate cancer cells through sustained activation of Erk1/2, c-jun/c-fos-dependent upregulation of p21, and induction of G(2) cell-cycle arrest. Cell Death Differ.

[32] Adamsen BL, Kravik KL, De Angelis PM (2011). DNA damage signaling in response to 5-fluorouracil in three colorectal cancer cell lines with different mismatch repair and TP53 status. Int J Oncol.

[33] Sommer H, Santi DV (1974). Purification and amino acid analysis of an active site peptide from thymidylate synthetase containing covalently bound 5-fluoro-2'-deoxyuridylate and methylenetetrahydrofolate. Biochem Biophys Res Commun.

[34] Cohen SM, Lippard SJ (2001). Cisplatin: from DNA damage to cancer chemotherapy. Prog Nucleic Acid Res Mol Biol.

[35] Xie S, Tu Z, Xiong J, Kang G, Zhao L, Hu W, Tan H, Tembo KM, Ding Q, Deng X, Huang J, Zhang Q (2017). CXCR4 promotes cisplatin-resistance of non-small cell lung cancer in a CYP1B1-dependent manner. Oncol Rep.

[36] Bijnsdorp IV, Schwendener RA, Schott H, Fichtner I, Smid K, Laan AC, Schott S, Losekoot N, Honeywell RJ, Peters GJ (2011). Cellular pharmacology of multi- and duplex drugs consisting of ethynylcytidine and 5-fluoro-2'-deoxyuridine. Invest New Drugs.

[37] Bolger AM, Lohse M, Usadel B (2014). Trimmomatic: a flexible trimmer for Illumina sequence data. Bioinformatics.

[38] Dobin A, Davis CA, Schlesinger F, Drenkow J, Zaleski C, Jha S, Batut P, Chaisson M, Gingeras TR (2013). STAR: ultrafast universal RNA-seq aligner. Bioinformatics.

[39] Liao Y, Smyth GK, Shi W (2013). The Subread aligner: fast, accurate and scalable read mapping by seed-and-vote. Nucleic Acids Res.

[40] Love MI, Huber W, Anders S (2014). Moderated estimation of fold change and dispersion for RNA-seq data with DESeq2. Genome Biol.

[41] Huang da W, Sherman BT, Lempicki RA (2009). Systematic and integrative analysis of large gene lists using DAVID bioinformatics resources. Nat Protoc.

[42] Luo W, Friedman MS, Shedden K, Hankenson KD, Woolf PJ (2009). GAGE: generally applicable gene set enrichment for pathway analysis. BMC Bioinformatics.

[43] Reynolds CP, Maurer BJ (2005). Evaluating response to antineoplastic drug combinations in tissue culture models. Methods Mol Med.

[44] Luo W, Brouwer C (2013). Pathview: an R/Bioconductor package for pathway-based data integration and visualization. Bioinformatics.

