# Supplementary material for: The conjugated antimetabolite 5-FdU-ECyd and its cellular and molecular effects on platinum-sensitive vs. -resistant ovarian cancer cells in vitro
Source: Oncotarget. 2017 Aug 14;8(44):76935–48. doi: 10.18632/oncotarget.20260 (PMC5652753; doi:10.18632/oncotarget.20260)
Supplement: Supplementary file 1 [file oncotarget-08-76935-s001.pdf]

The conjugated antimetabolite 5-FdU-ECyd and its cellular and molecular effects on platinum-sensitive vs. -resistant ovarian cancer cells *in vitro*

SUPPLEMENTARY MATERIALS

Cell lines

The isogenic human OC cell lines A2780 (platinum-sensitive), A2780cis (platinum-resistant) were purchased from Sigma-Aldrich (Taufkirchen, Germany) and were cultured in RPMI1640 medium, supplemented with 2 mM glutamine, 10 % fetal calf serum, 100 U/ml penicillin and 100 mg/ml streptomycin (Thermo Fisher Scientific, Waltham, Massachusetts, USA). Every 2-3 passages, the medium of A2780cis cells was additionally supplemented with 1 µM cisplatin for one passage, in order to maintain their platinum-resistant characteristics. PA-I cells (cancer cells, derived from human teratocarcinoma at the ovary) were cultured in the same medium as reported above, with additional supplementation of Insulin at 9 µg/ml. Platinum-resistant and highly malignant Skov-3-IP OC cells, a well described derivative from commercially available Skov-3 cells, which were originally obtained by *in vivo* passaging of Skov-3 cells in immunodeficient nude mice [1, 2], were cultured in DMEM high glucose medium, supplemented with 2 mM glutamine, 10 % fetal calf serum, 100 U/ml penicillin and 100 mg/ml streptomycin, 1x MEM non-essential amino acid solution and 10 mM HEPES buffer solution (Thermo Fisher Scientific, Waltham (Massachusetts), USA). All cell lines were maintained in a humified incubator with 5 % CO_2 at 37 °C.

Quantitative analysis of γH2AX staining

In all assays, nuclei were stained with Hoechst 33342 (5 µg/ml in 1 % Normal Goat Serum / 0.3 % Triton X-100-DPBS). Image acquisition was performed using fluorescence microscopy (Zeiss Axiovert 200) with a 40x objective lens. 16-bit images were acquired at random, across the entire microscopy slide. Image analysis was performed with ImageJ (1.49v). γH2AX foci were counted automatically, using the "find maxima macro" of ImageJ. Therefore, the area of the nucleus was traced using the Hoechst 33342 fluorescence signal. For γH2AX foci counting, an Alexa Fluor 488 intensity of 35 (A2780 cells and A2780cis) or 70 (Skov-3-IP) was set as threshold, tolerating ≤ 2 foci in apparently γH2AX-negative control cells. According to this threshold, a hundred randomly selected cells were counted for γH2AX foci in each condition. For combined γH2AX and cell cycle analysis, cells were co-stained with EdU, which marks proliferating cells in the S-phase. EdU-positivity was defined as an Alexa Fluor 555 intensity ≥ 100. γH2AX foci were counted in 10 EdU-positive vs. 10 EdU-negative cells. Experiments were performed in technical triplicates and statistical analysis was done with Prism 6.04 (GraphPad Software, CA, USA) using the unpaired t-test.

Drug interaction analysis

Drug interaction analysis between 5-FdU-ECyd and cisplatin was based on cell viability data, obtained by the fluorometric Cell Titer Blue®-Assay (Promega, Fitchburg, USA), according to the manufacturer's instructions. Statistical analysis was performed by the combination index method [3], which is based on the combination of drugs at a broad range of equipotent molar concentrations or, as our modification, with standard cisplatin dose-response curves with a fixed 5-FdU-ECyd concentrations at each reading point. For each combination, a combination index (CI) was calculated, in order to describe the drug interactions dynamics of 5-FdU-ECyd and cisplatin across a broad range of concentrations. A CI-value < 0.9 indicates a synergistic interaction, a CI-value between 0.9 and 1.1 indicates an additive interaction and a CI-value > 1.1 indicates an antagonistic interaction [3]. Log (Dose) vs. response curves (variable slope) were drawn with Prism 6.04 (GraphPad Software, CA, USA).

RNA-Seq read mapping and differential expression

Raw reads from Illumina sequencers were converted from bcl to fastq format using bcl2fastq (version v2.17.1.14) allowing for 1 barcode mismatch. Reads were trimmed for quality, sequence adapters and cropped to 75nt using trimmomatic [4] with the following parameters: TruSeq3-PE.fa:2:30:10:2:true LEADING:15 TRAILING:15 SLIDINGWINDOW:4:15 MINLEN:36 CROP:75. Trimming resulted in an average of 18 Mio reads per sample (maximum 23.4 Mio, minimum 11.7 Mio).

Reads were aligned to the Gencode genome (GRCh38.p7 primary assembly) using STAR [5] in a 2-pass mapping mode: first, an index was created using the genome sequence and gene annotation (here, Gencode GRCh38.p7 comprehensive gene annotation), against which all reads are aligned.

Second, all detected splice junctions of all samples are merged and used as guide for the second mapping step. The following parameters were used in both steps: --readFilesCommand zcat --alignIntronMax 500000 --alignMatesGapMax 500000 --outSAMtype BAM SortedByCoordinate --outSAMprimaryFlag OneBestScore --outFilterMultimapNmax 100 --outFilterMismatchNmax 2 --alignSJstitchMismatchNmax 5 -1 5 5. For the second step, additional parameters are: --sjdbFileChrStartEnd allSJ.out.tab --limitSjdbInsertNsj 100000000, where allSJ.out.tab denotes the collected splice junctions.

Read counts of all annotated genes were extracted from the alignments using featureCounts method of the Rsubread package [6] with the following parameters: GTF.featureType = "exon", GTF.attrType="gene_id", useMetaFeatures = T, isPairedEnd=T, requireBothEndsMapped=F, allowMultiOverlap=T, countMultiMappingReads=T, fraction=T. lincRNA and rRNA genes, and genes with 0 counts for all samples were discarded.

DESeq2 [7] was used to find differentially expressed genes using standard parameters. Multiple testing correction was performed using the Benjamini-Hochberg algorithm. The differentially expressed genes were filtered using the adjusted p-value$\leq$0.05. Significantly upregulated and downregulated genes were chosen based on a $\log_2$ fold-change (FC) value of 1.5.

Clustering was done using Euclidean distance and complete linkage. For sample distance analysis, regularized–logarithm transformation (rlog) values from DESeq2 were used for calculation of the Euclidean distance of samples and distance was visualized using the R package ComplexHeatmap [8]. Principal components analysis was done using the R stats package [9]. Unsupervised hierarchical clustering was done using ComplexHeatmap with the 50 top ranking protein-coding genes based on absolute $\log_2$ FC in the A2780cis cell line.

Pathway analysis

Pathway analysis was done using three different methods. First, Gene Ontology (GO) and KEGG enrichment of lists of differentially expressed genes with absolute $\log_2$ FC > 1.5 were calculated using DAVID Bioinformatics Resource [10] based on Ensembl IDs. The background set consisted of all genes passed to DESeq2. Second, the R package gage [11] was applied on the $\log_2$ FCs of all genes for the different conditions using KEGG pathways. Third, the R package fgsea [12] was used for a full gene set enrichment analysis. $-\log_{10}$(p-value) * signum ($\log_2$ FC) was used as rank function and 100,000 permutations for p-value calculation of pathway enrichments. KEGG pathways were plotted using the R package pathview [13].

REFERENCES

1. Yu D, Wolf JK, Scanlon M, Price JE, Hung MC. Enhanced c-erbB-2/neu expression in human ovarian cancer cells correlates with more severe malignancy that can be suppressed by E1A. Cancer Res. 1993; 53:891-898.
2. Bai F, Feng J, Cheng Y, Shi J, Yang R, Cui H. Analysis of gene expression patterns of ovarian cancer cell lines with different metastatic potentials. Int J Gynecol Cancer. 2006; 16:202-209.
3. Reynolds CP, Maurer BJ. Evaluating response to antineoplastic drug combinations in tissue culture models. Methods Mol Med. 2005; 110:173-183.
4. Bolger AM, Lohse M, Usadel B. Trimmomatic: a flexible trimmer for Illumina sequence data. Bioinformatics. 2014; 30:2114-2120.
5. Dobin A, Davis CA, Schlesinger F, Drenkow J, Zaleski C, Jha S, Batut P, Chaisson M, Gingeras TR. STAR: ultrafast universal RNA-seq aligner. Bioinformatics. 2013; 29:15-21.
6. Liao Y, Smyth GK, Shi W. The Subread aligner: fast, accurate and scalable read mapping by seed-and-vote. Nucleic Acids Res. 2013; 41:e108.
7. Love MI, Huber W, Anders S. Moderated estimation of fold change and dispersion for RNA-seq data with DESeq2. Genome Biol. 2014; 15:550.
8. Gu Z, Eils R, Schlesner M. Complex heatmaps reveal patterns and correlations in multidimensional genomic data. Bioinformatics. 2016; 32:2847-2849.
9. Team RC. R: a language and environment for statistical computing. R Foundation for Statistical Computing, Vienna, Austria. http://www.R-project.org/.
10. Huang da W, Sherman BT, Lempicki RA. Systematic and integrative analysis of large gene lists using DAVID bioinformatics resources. Nat Protoc. 2009; 4:44-57.
11. Luo W, Friedman MS, Shedden K, Hankenson KD, Woolf PJ. GAGE: generally applicable gene set enrichment for pathway analysis. BMC Bioinformatics. 2009; 10:161.
12. Sergushichev A. An algorithm for fast preranked gene set enrichment analysis using cumulative statistic calculation. bioRxiv. 2016. https://doi.org/10.1101/060012.
13. Luo W, Brouwer C. Pathview: an R/Bioconductor package for pathway-based data integration and visualization. Bioinformatics. 2013; 29:1830-1831.

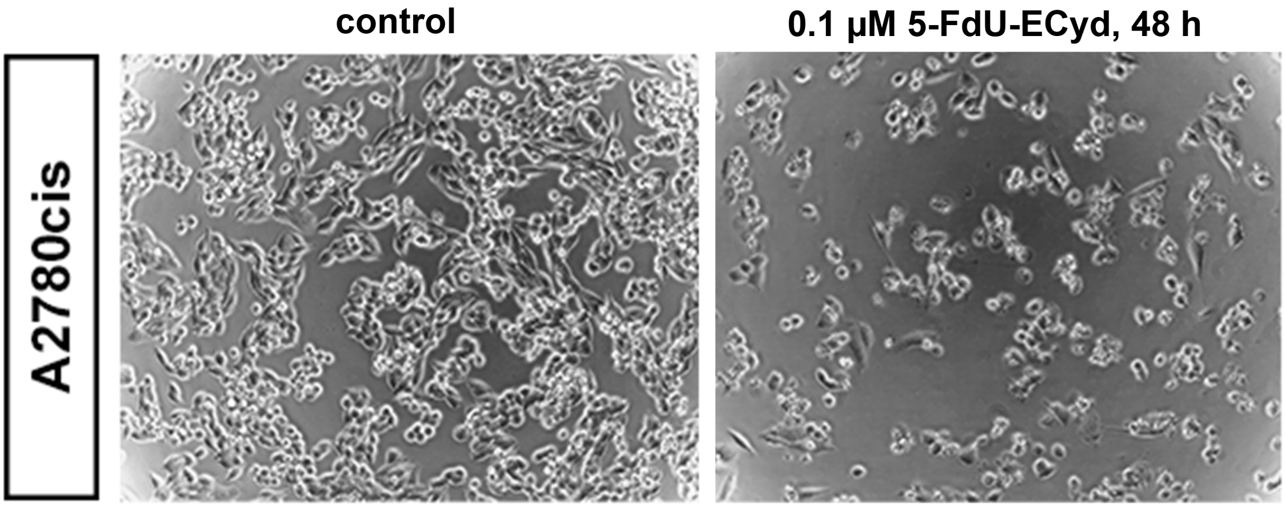

Supplementary Figure 1: The effect of 5-FdU-ECyd on the morphology of platinum-resistant ovarian cancer cells. The figure shows a representative example, how nanomolar 5-FdU-ECyd treatment (0.1 µM, 48 h) changes the morphology of platinum-resistant ovarian cancer cells (A2780cis). The confluency was clearly decreased, accompanied by morphological signs of apoptosis, such as cell fragmentation.

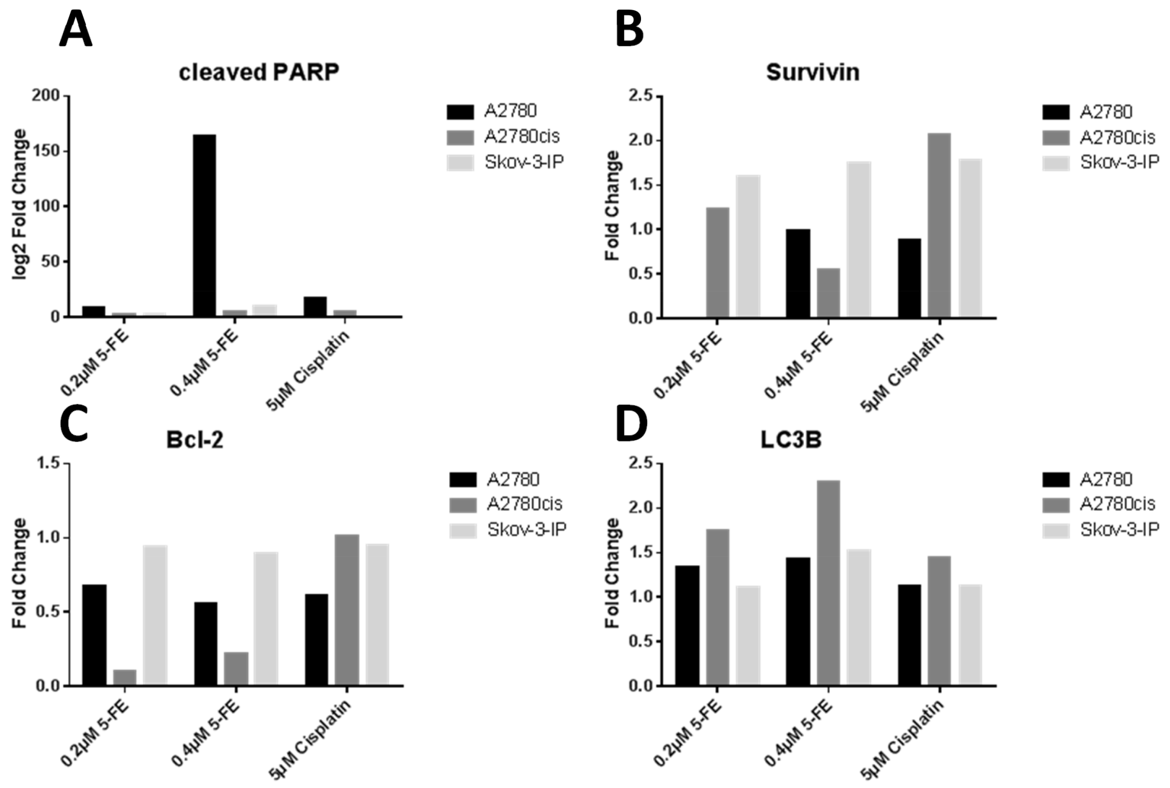

Supplementary Figure 2: Apoptosis and autophagy induction by 5-FdU-ECyd treatment. The bar charts show densitometric assessment of western blot analysis (Figure 1B in the manuscript). Calculated band density was normalized to ß-actin and is reported as fold change (or log2 fold change) in reference to untreated control.

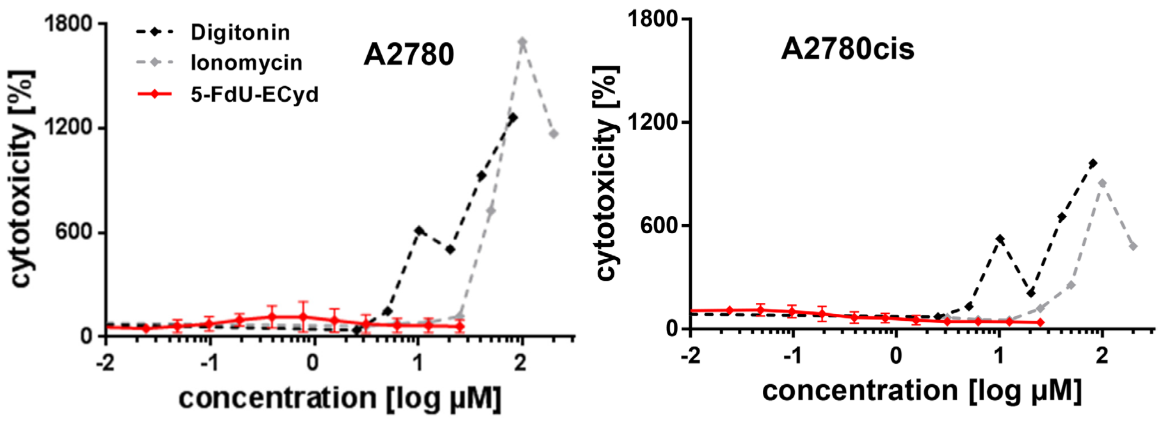

Supplementary Figure 3: Cytotoxicity assay of 5-FdU-ECyd treated ovarian cancer cells. The figure shows representative results from a cytotoxocity assay of platinum-sensitive A2780 and platinum-resistant A2780cis cells, following treatment across a broad range of 5-FdU-ECyd concentrations (0.024 µM – 25 µM, red dashed line). No evidence of cytotoxicity (primary necrosis) was oberved. Digitonin (black dashed line) and Ionomycin (grey dashed line) were used as positive control and show robust cytotoxicity induction in both cell lines. Normalized percentages of cytotoxicity in 5-FdU-ECyd treated cells were averaged from three independent experiments and are reported as mean ± SD.

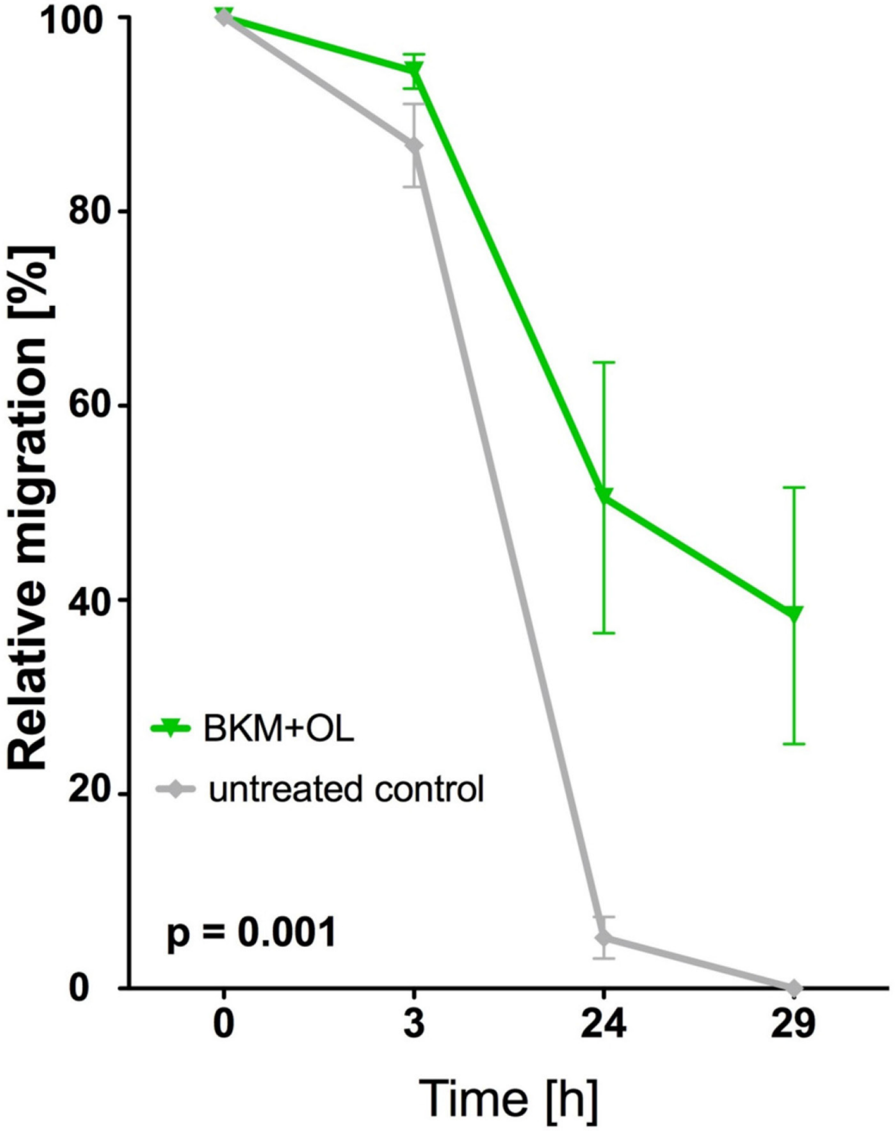

Supplementary Figure 4: Migration assay of 5-FdU-ECyd treated ovarian cancer cells. The figure shows the relative migration of platinum-resistant Skov-3-IP ovarian cancer cells after treatment with 1 µM BKM120 and 2 µM Olaparib (green line), compared to untreated control cells (grey line). Relative migration refers to the percentage of the cell free area over time. Values were calculated from three independent experiments and are reported as mean ± SEM. According to the two-way analysis of variance test (ANOVA), a statistically significant reduction in cell migration is shown after combined BKM120 and Olaparib treatment and therefore, this setting serves as positive control for this assay.

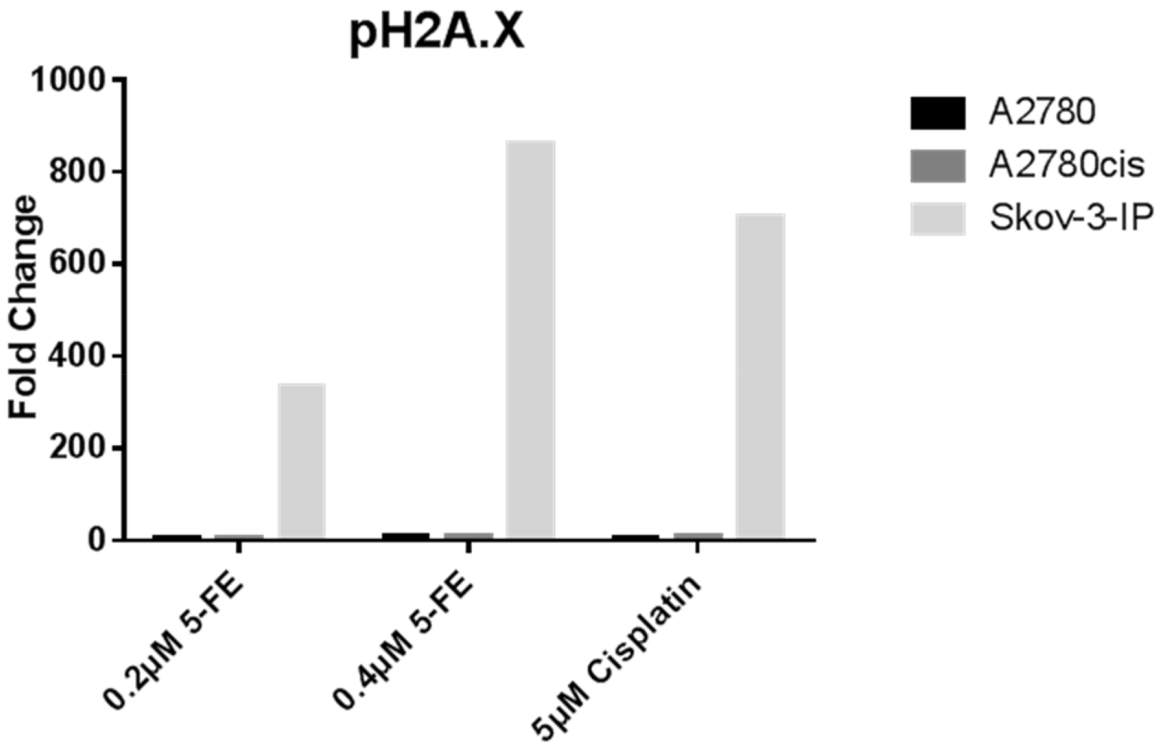

Supplementary Figure 5: DNA double strand break analysis in 5-FdU-ECyd treated cells. The bar chart shows densitometric assessment of western blot analysis (Figure 4A in the manuscript). Calculated band density was normalized to ß-actin and is reported as fold change in reference to untreated control.

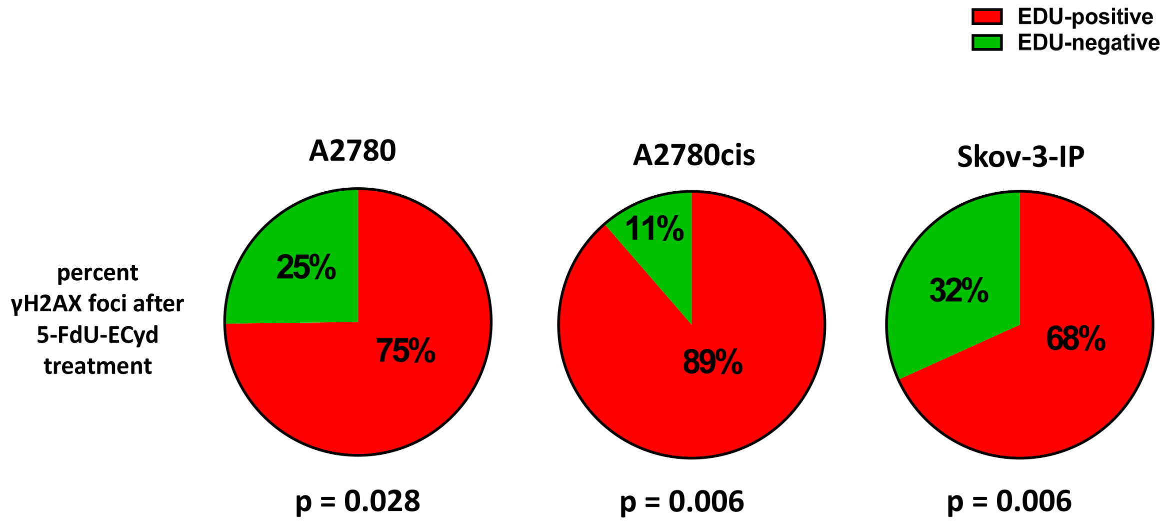

Supplementary Figure 6: Cell cycle dependence of γH2AX foci formation by 5-FdU-ECyd treatment in OC cells. The pie chart shows the percentage of γH2AX foci, following treatment with 5-FdU-ECyd (A2780 and A2780cis: 0.1 nM, Skov-3-IP: 0.4 nM), in EdU-positive S-phase cells vs. non-replicating EdU-negative cells. Statistical significance was calculated, according to the student's t-test.

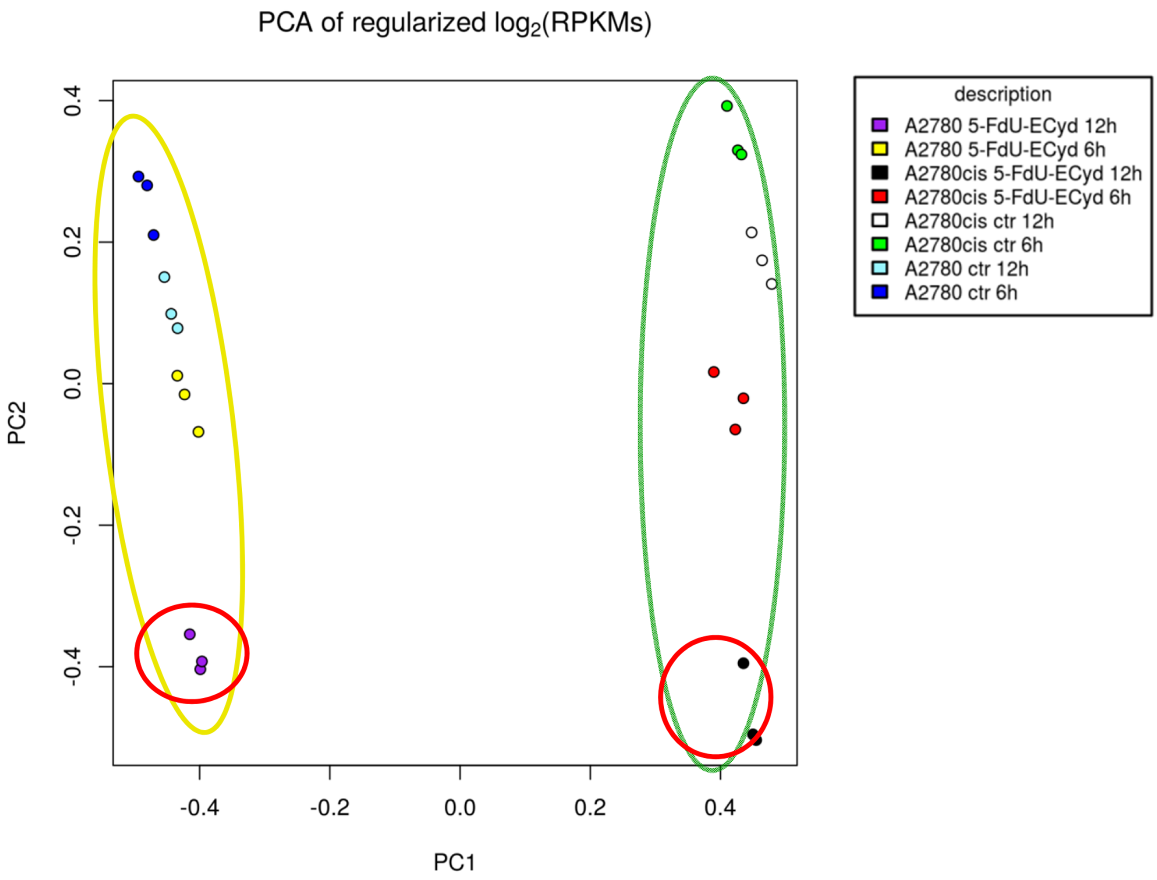

Supplementary Figure 7: Principal components analysis (PCA) of RNA-seq expression data in A2780 and A2780cis with and without 5-FdU-ECyd treatment shows clear grouping of samples into biological triplicates (color coded). PCA reveals clear separation of platinum-sensitive A2780 cells (yellow circle) and A2780cis cells (green circle) as well as marked separation after 5-FdU-ECyd treatment for 12 h in both cell lines (red circles).

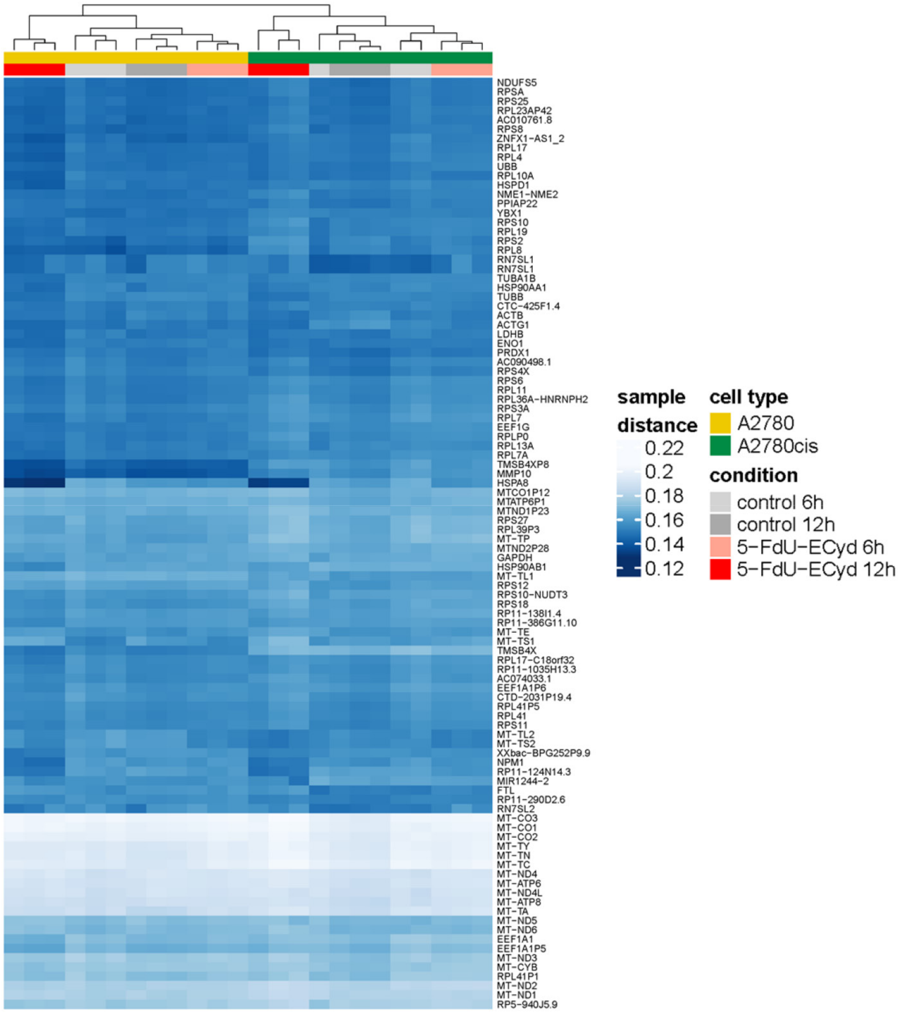

Supplementary Figure 8: Differential gene expression in 5-FdU-ECyd treated ovarian cancer cells. Heat map showing unsupervised hierarchical clustering using the top 100 expressed genes over all cell lines and conditions. The top color bar represents the cell line and the second color bar the treatment type (done in triplicates). Each column denotes a sample. Each row denotes a single gene and their expression pattern across the samples. The dendrogram on the x-axis shows the grouping of same samples. Samples cluster in two main groups according to the cell type (A2780 and A2780cis) and within both of these groups there is clear separation of cells treated with 5-FdU-ECyd for 12 h (dark red).

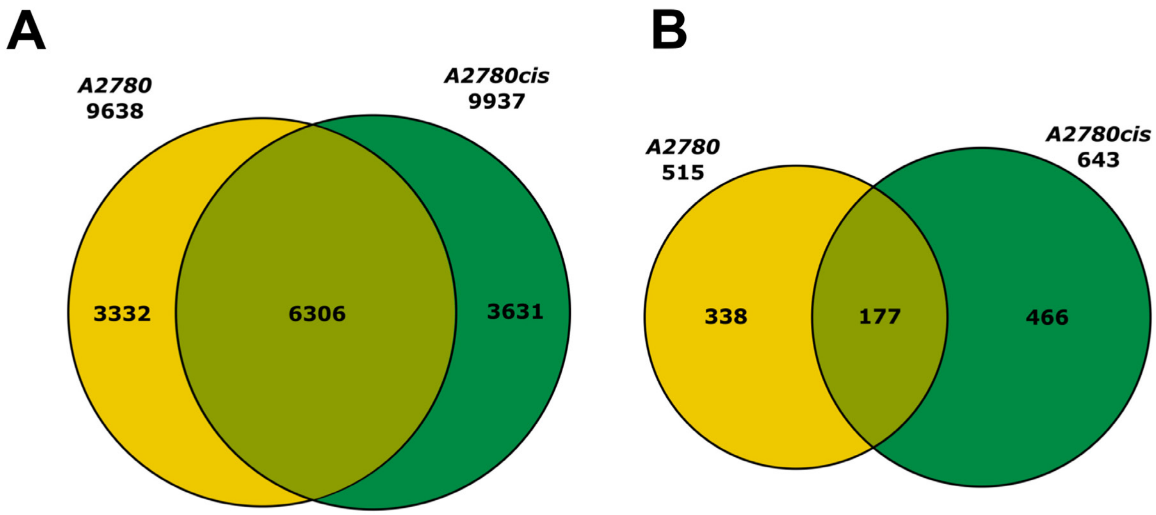

Supplementary Figure 9: Differentially expressed genes between 5-FdU-ECyd treated and untreated cells in A2780 and A2780cis ovarian cancer cell lines. The venn diagramme depicts the number of differentially expressed genes after 5-FdU-ECyd treatment for 12 h in platinum-sensitive A2780 cells (yellow circle) and platinum-resistant A2780cis cells (green circle) and the overlap of both cell lines (brown intersection shape). **(A)** Significantly differentially expressed genes based on p-value < 0.05 adjusted for multiple testing by Benjamini-Hochberg procedure. **(B)** Significantly differentially expressed genes (adjusted p-value < 0.05) with an absolute log2 fold-change ≥ 2.

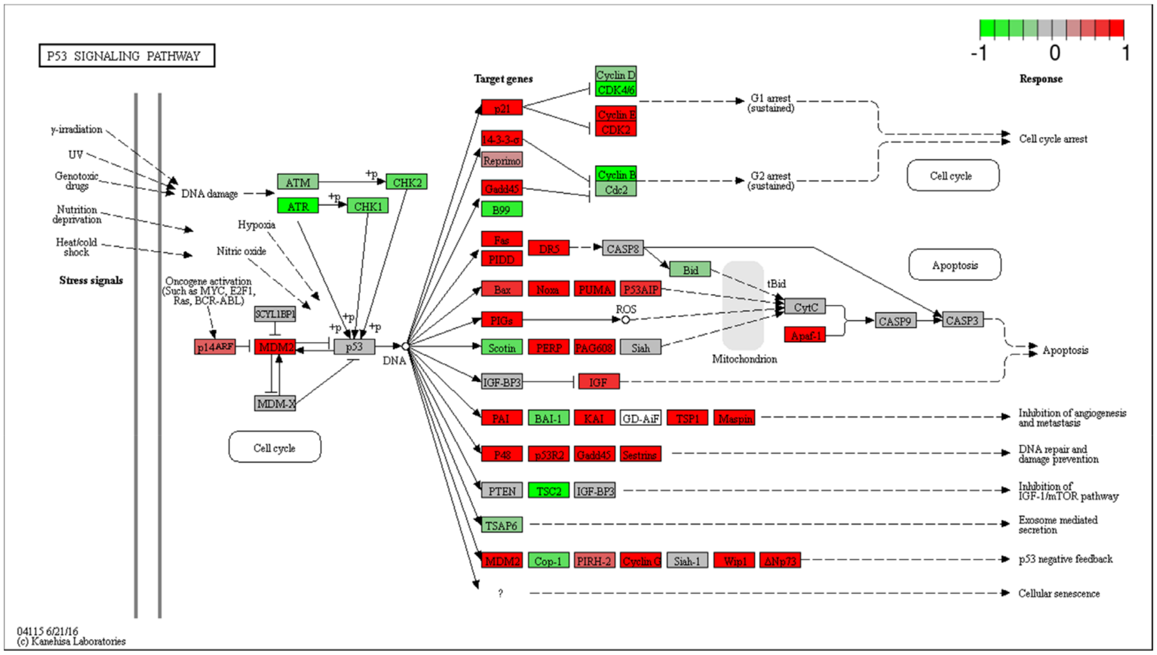

Supplementary Figure 10: Upregulation of the p53-pathway following 5-FdU-ECyd treatment of platinum-sensitive A2780 ovarian cancer cells. Plots showing the p53-pathway with genes color coded according to normalized log2 fold-changes after 12 h 5-FdU-ECyd treatment in A2780 platinum-sensitive ovarian cancer cells. KEGG pathways were plotted using the R package pathview.

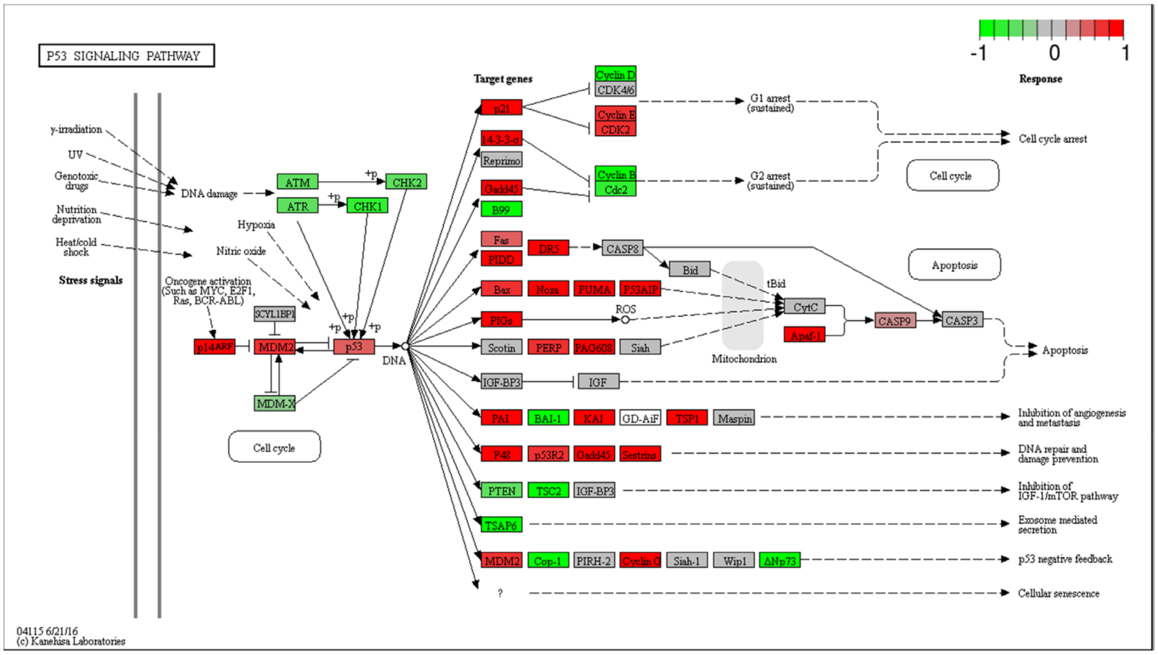

Supplementary Figure 11: Upregulation of the p53-pathway following 5-FdU-ECyd treatment of platinum-resistant A2780cis ovarian cancer cells. Plots showing the p53-pathway with genes color coded according to normalized log2 fold-changes after 12 h 5-FdU-ECyd treatment for A2780cis platinum-resistant ovarian cancer cells. KEGG pathways were plotted using the R package pathview.

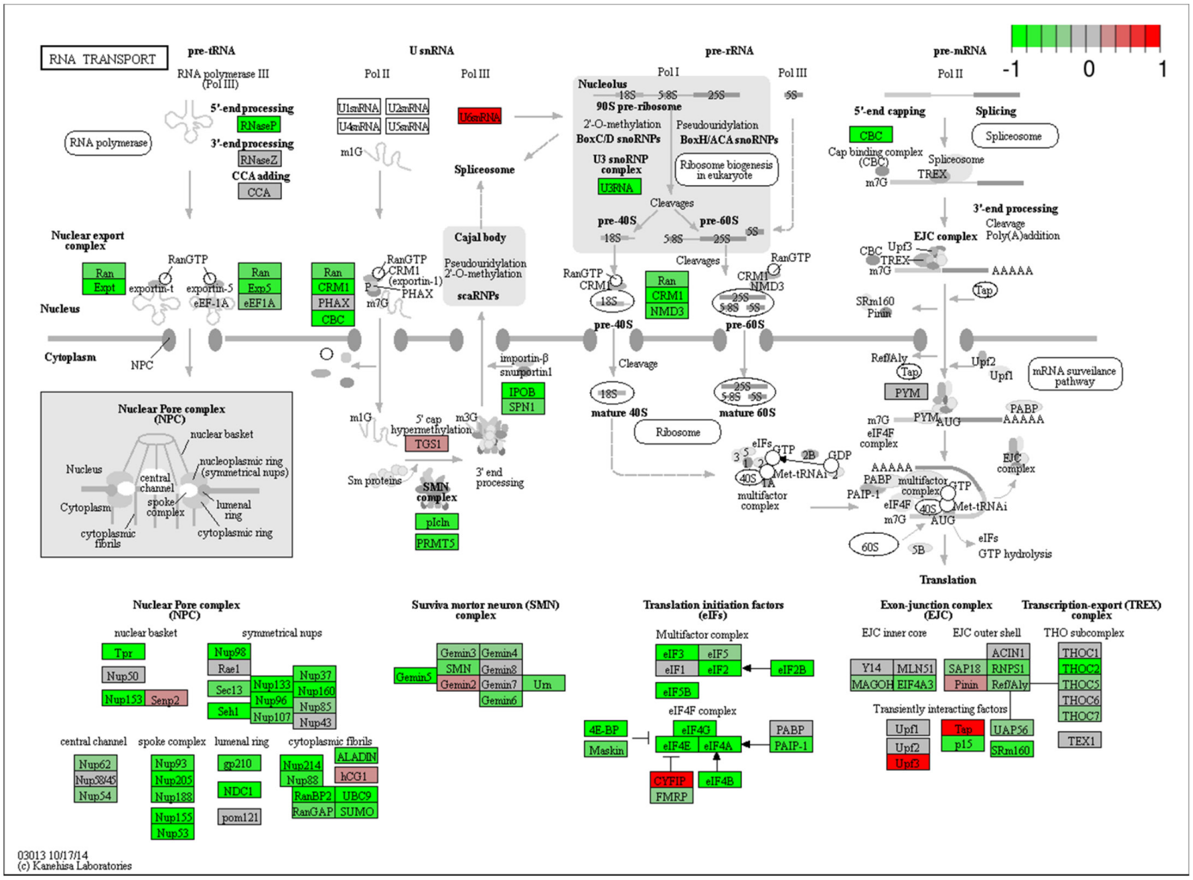

Supplementary Figure 12: Downregulation of the RNA transport following 5-FdU-ECyd treatment of platinum-sensitive A2780 ovarian cancer cells. Plots showing genes involved in RNA transport with genes color coded according to normalized log2 fold-changes after 12 h 5-FdU-ECyd treatment for platinum-sensitive A2780 ovarian cancer cells. KEGG pathways were plotted using the R package pathview.

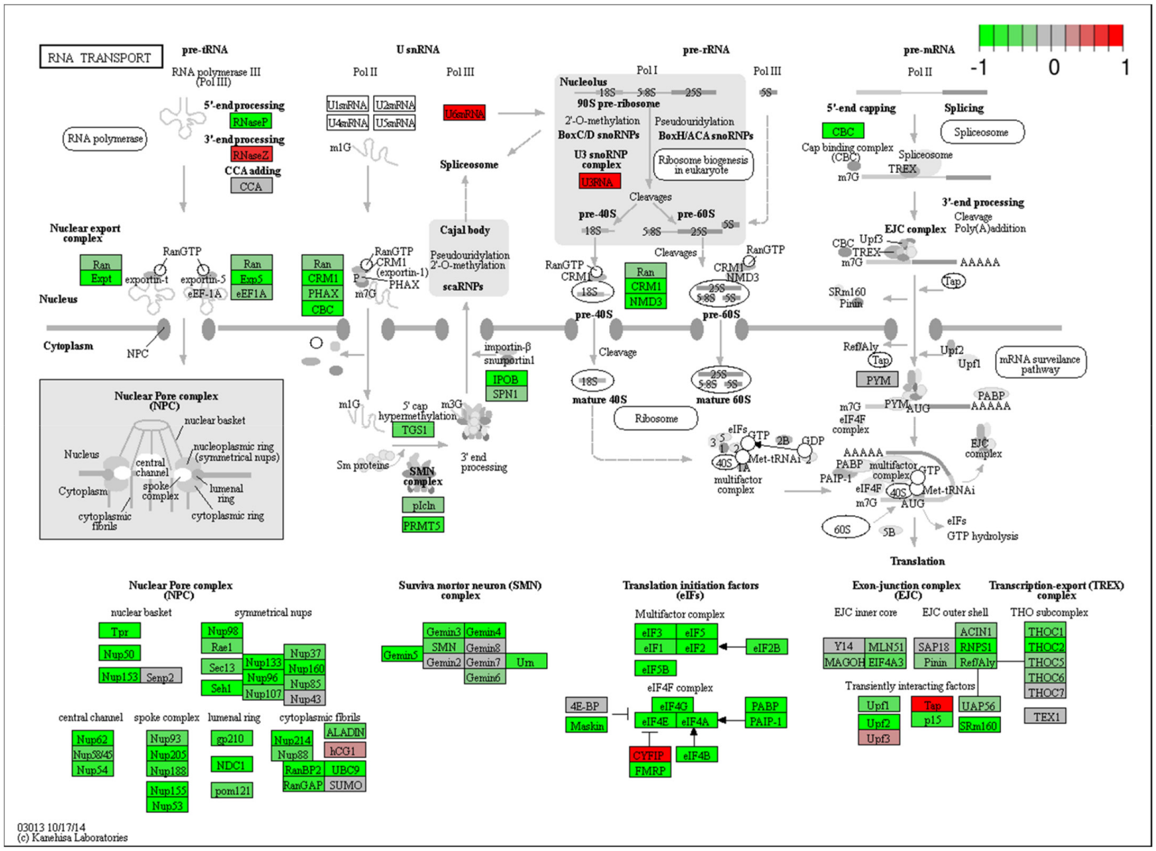

Supplementary Figure 13: Downregulation of the RNA transport following 5-FdU-ECyd treatment of platinum-resistant A2780cis ovarian cancer cells. Plots showing genes involved in RNA transport with genes color coded according to normalized log2 fold-changes after 12 h 5-FdU-ECyd treatment for platinum-resistant A2780 ovarian cancer cells. KEGG pathways were plotted using the R package pathview.
